# Current Advances in Black Phosphorus‐Based Drug Delivery Systems for Cancer Therapy

**DOI:** 10.1002/advs.202003033

**Published:** 2021-01-15

**Authors:** Wenxin Liu, Alideertu Dong, Bing Wang, Han Zhang

**Affiliations:** ^1^ College of Chemistry and Chemical Engineering Inner Mongolia University Hohhot 010021 P. R. China; ^2^ Engineering Research Center of Dairy Quality and Safety Control Technology Ministry of Education Inner Mongolia University Hohhot 010021 P. R. China; ^3^ Shenzhen Engineering Laboratory of Phosphorene and Optoelectronics Key Laboratory of Optoelectronic Devices and Systems of Ministry of Education and Guangdong Province College of Physics and Optoelectronic Engineering Shenzhen University Shenzhen 518060 P. R. China

**Keywords:** biocompatibility, black phosphorus, cancer therapy, drug delivery, stimuli‐responsive release

## Abstract

Cancer has been one of the major threats to the lives of human beings for centuries. Traditional therapy is more or less faced with certain defects, such as poor targeting, easy degradation, high side effects, etc. Therefore, in order to improve the treatment efficiency of drugs, an intelligent drug delivery system (DDS) is considered as a promising solution strategy. Due to their special structure and large specific surface area, 2D materials are considered to be a good platform for drug delivery. Black phosphorus (BP), as a new star of the 2D family, is recommended to have the potential to construct DDS by virtue of its outstanding photothermal therapy (PTT), photodynamic therapy (PDT), and biodegradable properties. This tutorial review is intended to provide an introduction of the current advances in BP‐based DDSs for cancer therapy, which covers topics from its construction, classified by the types of platforms, to the stimuli‐responsive controlled drug release. Moreover, their cancer therapy applications including mono‐, bi‐, and multi‐modal synergistic cancer therapy as well as the research of biocompatibility are also discussed. Finally, the current status and future prospects of BP‐based DDSs for cancer therapy are summarized.

## Graphical abstract

In order to solve the drawbacks of traditional treatment methods, drug delivery systems (DDSs) based on 2D materials, especially black phosphorus (BP), are continuously explored. Current advances of BP‐based DDSs in cancer therapy, from construction, drug release, cancer therapy, and biocompatibility, are reviewed, and the current status and future outlook are also discussed.

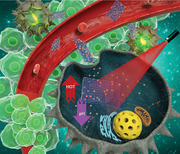

## 1 Introduction

As one of the world's deadliest diseases, cancer is feared all over the globe. To date, huge amounts of material, financial, and human resources have been invested in the field of tumor diagnosis. Various medical imaging technologies, such as magnetic resonance imaging (MRI), ultrasound, and computed tomography have been successfully applied in clinical practice, increasing our understanding, discovery, and diagnosis of tumors.^[^ ^1^, ^2^, ^3^, ^4^, ^5^ ^]^ However, due to the variability and complexity of tumor cells, treatment, especially of malignant tumors, still faces multiple significant challenges. Current clinical therapies to combat cancer primarily involve surgery, chemotherapy, and radiotherapy (RT). But surgery has the disadvantage of incomplete clearance, and the low efficiency of chemotherapy and side effects of radiotherapy limit their applications. Up‐and‐coming tumor treatments include photothermal therapy (PTT), photodynamic therapy (PDT), and gene therapy (GT), but these are still in the experimental stages.^[^ ^6^ ^]^

Recently, 2D materials such as graphene oxide (GO), black phosphorus (BP), molybdenum disulfide are being extensively studied for use in cancer therapy.^[^ ^7^, ^8^, ^9^, ^10^ ^]^ Novel 2D monoelemental materials, in particular (Xenes, such as borophene, gallenene, silicene, germanene, stanene, phosphorene, arsenene, antimonene, bismuthene, tellurene, and selenene), have shown remarkable potential for their applications in different fields.^[^ ^11^ ^]^ In addition to having tunable layer‐dependent bandgaps, Xenes are also superior to other 2D materials in the ease with which they can be eliminated and degraded in biological systems. Combined with their tractability for synthetic exploration, these qualities have led to the broad application of Xenes in various biomedical fields.^[^ ^11^ ^]^ For example, germanene‐based theranostic materials in the form of nanosheet‐DDS or hydrogel‐DDS have exhibited high drug‐loading capacity, controlled drug release, and efficient inhibition of tumor recurrence and wound infection.^[^ ^12^ ^]^ Similar, germanene quantum dots and antimonene quantum dots have also shown excellent PTT ability in the hyperpyrexia ablation of tumors.^[^ ^13^, ^14^ ^]^ BP in particular is a rising star in the 2D mono‐elemental family, and in recent years, its high hole mobility, adjustable bandgap, and strong optical absorption have attracted attention and led to studies in numerous fields, such as optical sensing, photodetectors, pohotocatalysts cancer therapy etc.^[^ ^15^, ^16^, ^17^ ^]^ As a semiconducting material, its ultrahigh charge mobility, small electric resistance, wide photon absorption window, and in‐plane anisotropy suggest its potential as a photocatalyst.^[^ ^18^, ^19^ ^]^ Its bandgap of 0.3–2 eV from bulk to monolayer makes BP excellent for use in mid‐IR photonic devices as well as high‐performance field‐effect transistors.^[^ ^20^, ^21^, ^22^ ^]^

In the past few years, these characteristics have led BP to be developed for a wide range of practical application in batteries, electronics, sensors, and catalysts, which have been reported and summarized in many reviews.^[^ ^23^, ^24^, ^25^, ^26^ ^]^ The practical application of BP in electronic components is limited by its instability under certain conditions, which often leads to the inactivation of components.^[^ ^27^, ^28^, ^29^, ^30^ ^]^ But the environmental instability of BP may be an advantage for in vitro and in vivo biomedical applications, as it is highly biodegradable and therefore appropriate for use in biomaterials. Indeed, these are the unique advantages of BP over other 2D materials in developing DDSs for cancer therapy.^[^ ^31^, ^32^, ^33^ ^]^ Phosphorus is a vital element for living organisms and constitutes ≈1% of the human body's total weight. BP degrades into harmless phosphate in physiological environments, giving it high biocompatibility and low cytotoxicity compared with other 2D materials.^[^ ^34^ ^]^ BP's degradability in physiological environments prevents it from accumulating in vivo, making it a highly biocompatible material.

In 2015, BP was shown to be an effective PTT agent due to its high near‐infrared (NIR) photothermal conversion efficiency.^[^ ^35^ ^]^ In the same year, exfoliated BP was shown to be an effective photosensitizer (PS) for the generation of singlet oxygen (SO), with a high quantum yield of about 0.91, making it attractive for use in PDT.^[^ ^36^ ^]^ Given BP's good biocompatibility and excellent PTT and PDT capabilities, BP nanomaterials have attracted enormous attention in biomedical applications, and developments have multiplied in recent years.^[^ ^37^, ^38^, ^39^, ^40^ ^]^ In addition, the large surface area of BP and its fold structure result in large numbers of anchor points for guest therapeutic agents such as anticancer drugs, pointing to its eligibility for use in drug delivery systems (DDSs).

To our knowledge, BP nanosheets (BPNs) loaded with doxorubicin (DOX) were the first BP‐based DDS for synergistic PDT/PTT/chemotherapy to treat cancer, reported in 2017.^[^ ^41^ ^]^ In recent years, the limitations of bare BPNs, such as instability and insufficient numbers of active sites, have promoted the development of modified BPNs. Black phosphorus quantum dots (BPQDs) and black phosphorus hydrogels (BPHs), both common forms of the BP family, have also been introduced as platforms due to their unique advantages, which include high photothermal conversion efficiency,^[^ ^35^ ^]^ high water content, minimal invasiveness, and biocompatibility,^[^ ^42^, ^43^, ^44^ ^]^ broadening the practical scope of BP‐based DDSs. Various drugs have been used with these platforms, from the most common chemotherapy drugs like DOX, to paclitaxel (PTX),^[^ ^45^ ^]^ bortezomib (BTZ),^[^ ^46^ ^]^ cisplatin, oxaliplatin,^[^ ^47^ ^]^ and even traditional Chinese medicines (e.g., kirenol (KIR)).^[^ ^48^ ^]^ Some nanoparticles (NPs) and upconversion nanoparticles (UCNP) have also been successfully constructed for BP‐based DDSs.^[^ ^49^, ^50^, ^51^, ^52^, ^53^ ^]^ Such rapid development of novel therapies should lead to the enrichment of monomodal cancer therapies such as PDT, PTT, and GT, or to improvements in multimodal therapies.^[^ ^54^, ^55^, ^56^, ^57^, ^58^, ^59^ ^]^ Multifunctional DDSs that can be used with surface‐enhanced Raman scattering (SERS), MRI, or polyethyleneimine (PEI) imaging also represent further progress in drug delivery targeting and tracking.^[^ ^49^, ^50^, ^60^ ^]^

Clearly, the entire field is too large to be covered exhaustively here, but in view of the increasingly important application value of BP, we undertake to highlight recent studies of BP‐based DDSs for cancer therapy. Some relevant reviews have been published on the biomedical applications of BP, including their preparation, functionalization, and applications (imaging, biosensors), as well as an overall review of the application of BP in cancer therapy.^[^ ^34^, ^39^, ^61^, ^62^, ^63^, ^64^ ^]^ But in each instance, only part of the review is related to cancer therapy, and the coverage is not comprehensive or sufficiently detailed. The present review first provides a comprehensive summary of recent advances in BP‐based DDSs, especially the construction of BP‐based DDSs and the achievement of stimuli‐responsive release, a topic thus far rarely reviewed. We discuss the construction of BP‐based DDSs, classified according to BP platform type (bare BPNs, modified BPNs, BPQDs, and BPHs). In particular, substrates, loaded drugs, and the combination of forces during the construction of DDSs are described and compared to facilitate customization for actual requirements. We then present in detail stimuli‐responsive types of controlled drug release, and highlight applications in cancer therapies, including mono‐, bi‐, and multimodal synergistic cancer therapies that have caught the interest of the scientific community. Finally, we review how the biocompatibility of DDSs is assessed using in vitro and in vivo methods.

## 2 Construction of BP‐Based DDSs

**Figure 1:**
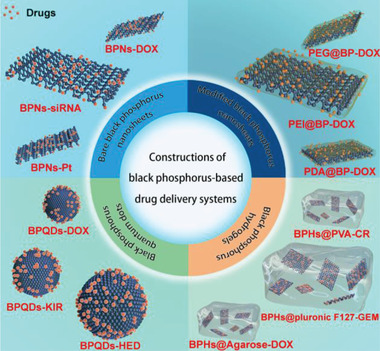
Schematic diagram of the four types of platforms of the construction of BP‐based DDSs.

BP holds significant promise for use in DDSs due to its high specific surface‐to‐volume ratio, photosensitivity, broad light absorption, excellent biocompatibility, and high biodegradability. The main categories of BP platforms are classified in four types: bare BPNs, modified BPNs, BPQDs, and BPHs, which are schematized in **Figure** **1**. Using BP as an effective carrier, a wide variety of DDSs have been developed by loading drugs that include common clinical anticancer drugs (such as, DOX, PTX, and BTZ), small interfering RNA (siRNA), inorganic components (e.g., Au, Fe_3_O_4_, Pt, and UCNP), and others. These BP‐based DDSs fight cancer in different ways, with some showing a single anticancer effect, while others combine multiple features, such as imaging and biodetection.^[^ ^50^, ^65^ ^]^

To date, three major strategies have been explored for constructing BP‐based DDS platforms: electrostatic interaction, covalent bonding, and noncovalent bonding (e.g., hydrophobic interactions). Since BP carries a negative charge, positively charged drugs can easily be loaded onto it via electrostatic interaction. DDSs can also be fabricated by bonding drugs onto a BP delivery platform. They can be classified according to the form of BP being used, as summarized in **Table** **1**.

**Table 1:** Classification and characteristics of BP‐based DDSs

Platforms	Coating	Bonding ways	Payloads	Tumor cells	Remarks	Refs.
Black phosphorus nanosheets	None	Electrostatic interaction	Doxorubicin	4T1 tumors	Due to their negative charge and corrugated surface structure, bare BPNs can easily be loaded with small, positively charged drugs via electrostatic interaction. Ones that are neutral or negatively charged can also be loaded after the drugs have been electrically modified. However, this method does not work with drugs that are difficult to electrically modify directly; the strength of electrostatic interaction is not always strong enough and is easily affected by pH, electrical fields, and other factors, leading to low drug loading, imprecise release, and suboptimal efficiency.	^[^ ^41^ ^]^
				MCF‐7 cells		^[^ ^46^, ^66^ ^]^
				MCF‐7 cells, resistant MCF‐7 cells		^[^ ^67^ ^]^
			Mitoxantrone hydrochloride	4T1 tumor		^[^ ^68^ ^]^
			Cas9–sgRNA	MCF‐7 cells, A549 tumor		^[^ ^56^ ^]^
			Au	HeLa cells		^[^ ^49^ ^]^
			Fe_3_O_4_	HeLa cells		^[^ ^49^ ^]^
		In situ reduction	Au	4T1 tumor	In situ reduction is a widespread strategy requiring no additional reducing agent because of the excellent reducing capability of BPNs, caused by their high Fermi level, but they are always susceptible to aggregation and settling in real‐world use.	^[^ ^50^ ^]^
				Hep G2 cells, 4T1 tumor		^[^ ^70^ ^]^
			Pt	4T1 tumor		^[^ ^51^ ^]^
		Covalent bonding	Nile blue	MCF‐7 cells	Chemical bonds such as covalent bonds, coordinate bonds, *π*‐bonding, and so on have also been adopted to construct BPN‐based DDSs with a stronger bonding energy. However, this often requires complex organic reactions and a high degree of purity.	^[^ ^65^ ^]^
		Coordinate bonding	Cisplatin, Oxaliplatin	Ovarian cell line A2780		^[^ ^47^ ^]^
			DACHPt	HeLa cells		^[^ ^71^ ^]^
Modified black phosphorus nanosheets	Polyethylene glycol‐amine	Electrostatic interaction	Upconversion nanoparticles	HeLa cells, U14 cells	BPNs modified with PEG–NH_2_ via electrostatic adsorption have exhibited good biocompatibility and physiological stability, with almost no aggregation or degradation. The amino groups can also act as functional groups for further reaction with loaded drugs.	^[^ ^52^ ^]^
			Doxorubicin	HeLa cells		^[^ ^42^, ^72^ ^]^
		Conjugate	Cyanine7	4T1 tumor		^[^ ^253^ ^]^
		Covalent bonding	Chlorin e6	HeLa cells		^[^ ^44^ ^]^
			Upconversion nanoparticles	HeLa cells		^[^ ^53^ ^]^
	Polyethylenimine	Electrostatic interaction	siRNA	HeLa cells, A549 tumor	PEI has abundant –NH_2_ groups, making it able to neutralize almost any negatively charged substance. It is easily combined with BP and hence widely used to change the electrical properties of the substrate in a BP‐based drug delivery system.	^[^ ^55^ ^]^
			siRNA	MCF‐7 cells		^[^ ^57^ ^]^
			Au	Hep G2 cells		^[^ ^73^ ^]^
	Polydopamine	Covalent bonding	Chlorin e6	HeLa cells	PDA is a well‐known biomimetic polymer with high adhesive capacity that is easily synthesized in an alkaline environment. In BP‐based DDSs, PDA coating also confers enhanced stability and photothermal effects.	^[^ ^75^ ^]^
			Bortezomib	MCF‐7 cells		^[^ ^46^ ^]^
	Poly(2‐ethyl‐2‐oxazoline)	Covalent bonding	Bortezomib	MCF‐7 cells	PEOz has high water solubility, flexibility, and biocompatibility. Its unique charge‐reverse capability in low pH makes it an ideal pH‐responsive drug release modification for DDSs.	^[^ ^46^ ^]^
	Human serum albumin	Hydrophobic interactions	Paclitaxel	U87MG cells	Prepared BP–HSA exhibits excellent biodegradability and biocompatibility, allowing it to play a triple role: exfoliant, capping agent, and drug carrier.	^[^ ^45^ ^]^
Black phosphorus quantum dots	Polyelectrolyte polymers	Electrostatic interaction	siRNA	PA‐1 cells	The high photothermal conversion efficiency of BPQDs makes them excellent in PTT therapy. When BPQDs bind to positively charged components through electrostatic interactions, the resulting changes in electrical properties improve biocompatibility, increase the number of functional groups, and confer targeted functionality. Another strategy is to introduce a capsule structure using a biofilm on the outside of the BPQDs, which acts to encapsulate them and prevent leakage, trapping the drug and BPQDs together within the membrane. Compared with nanosheets, the smaller size and higher specific surface area of a BPQD platform can allow better absorption by the body, free travel within blood vessels, and enhanced permeability and retention for targeted delivery. However, the BPQD system has several disadvantages: it is difficult to operate, medicine delivery is challenging, and it is apt to discharge.	^[^ ^54^ ^]^
	Polyethylene glycol‐amine	Covalent bonding	Doxorubicin	HeLa cells		^[^ ^81^ ^]^
		Electrostatic interaction	Rhodamine B	4T1 tumor		^[^ ^80^ ^]^
	Mesoporous silica framework	In situ reduction	Pt	Hep G2 cells		^[^ ^59^ ^]^
	None	Hydrogen bonds or electrostatic forces	Hederagenin	MCF‐7 cells		^[^ ^82^ ^]^
			Doxorubicin	HeLa cell		^[^ ^48^ ^]^
			Kirenol	HeLa cells, Raw264.7 cells		^[^ ^48^ ^]^
Black phosphorus hydrogels	Pluronic F127	Viscosity of the hydrogels	Gemcitabine	4T1 tumor	Hydrogels are considered excellent biological materials because of their special structure, high water content, minimal invasiveness, and biocompatibility. Phase transition in thermosensitive hydrogels is very flexible and easily reversed by external conditions, increasing the materials’ potential for use in controlled drug release. However, hydrogels are usually limited to the epidermis and have a narrow range of application in vivo.	^[^ ^93^ ^]^
	Agarose	Viscosity of the hydrogels	Doxorubicin	MDA–MB–231 tumors		^[^ ^92^ ^]^

### 2.1 Bare BPNs Platform

Since the first exfoliation of BP in 2014, BPNs have become the most popular 2D BP materials and are widely used as a substrate in drug delivery. Due to their negative charge and corrugated surface structure, bare BPNs can easily be loaded with small, positively charged drugs via electrostatic interaction between the support and the drug. Chen et al. reported a DDS for synergistic combination therapy based on electrostatic interaction between BPNs and DOX, with a high DOX loading capacity of 950 wt%.^[^ ^41^ ^]^ In Gao et al.’s two studies,^[^ ^46^, ^66^ ^]^ the DOX loading of 300% was achieved on bare BPNs, and Wu et al.’s study attained 233% loading.^[^ ^67^ ^]^ The as‐prepared BPNs‐DOX greatly improved the transport efficiency of DOX in vivo, reduced drug loss before activity began, and improved biocompatibility and antitumor efficiency. In addition to DOX, mitoxantrone hydrochloride (MTX), an effective clinical cationic drug, can be absorbed onto BPNs via electrostatic interaction, as described in Zhang et al.’s report.^[^ ^68^ ^]^ This DDS significantly inhibited tumor growth and was markedly superior to single‐treatment MTX chemotherapy, showing great potential for use in antitumor therapy.

With the help of a BPNs carrier, traditional anticancer drugs achieve higher efficiency in chemotherapy, demonstrating the potential of BPNs as a drug delivery substrate. As a result, DDSs based on BPNs have gradually been extended to the field of GT. For example, Zhou et al. constructed a BPN‐supported GT platform via a two‐step synthesis process.^[^ ^56^ ^]^ First, Cas9 protein was assembled with three repeated nuclear localization signals (NLSs). Because of the positively charged basic amino acid residues in the NLSs, the as‐assembled Cas9–sgRNA complexes (Cas9N3) could then be loaded onto the BPNs via electrostatic interaction, achieving a remarkable loading capacity of 98.7%. This gene–protein combined carrier system widens the range of BPNs as carriers, and enables active molecules and macromolecules to be used in BPN‐based DDSs via electrostatics.

**Figure 2:**
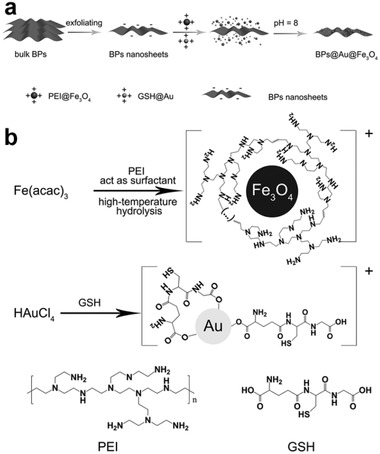
a) Schematic illustration for the fabrication of BPs@Au@Fe_3_O_4_ nanoplatform. b) The formation illustration of two important precursors. Reproduced with permission.^[^ ^49^ ^]^ Copyright 2017, Wiley‐VCH.

In addition to drugs with positive charges, ones that are neutral or negatively charged can also be loaded onto BPNs after the drugs have been electrically modified. This versatility makes BPNs more attractive than other substrates for developing DDSs. Of the various methods that effectively immobilize neutral or negative drugs onto BPNs, the polymer coating strategy is the most popular. Yang et al. deposited both Au and Fe_3_O_4_ NPs on BPNs to achieve three purposes: a photothermal effect, tumor targeting, and imaging.^[^ ^49^ ^]^ As seen in **Figure** **2**, Au nanoparticles were prepared by coordinating Au ions with the carboxylate groups of glutathione (GSH), and Fe_3_O_4_ was obtained via a high‐temperature hydrolysis reaction with PEI as a surfactant. The introduction of GSH and PEI enabled the Au NPs and Fe_3_O_4_ NPs to effectively adsorb onto the surface of the BPNs. A modification strategy such as this can be extended to other nonpositively charged chemicals, opening a new avenue for the construction of DDSs based on bare BPNs.

It is straightforward and convenient to build DDSs through electrostatic interaction, but this method does not work with drugs that are difficult to electrically modified directly. In addition, the strength of electrostatic interaction is not always strong enough and is easily affected by pH, electrical fields, and other factors. So enhancing the binding ability between BPNs and drugs is a better tactic for developing stable BPN‐based DDSs.

In situ reduction is a widespread strategy requiring no additional reducing agent because of the excellent reducing capability of BPNs caused by their high Fermi level, which is calculated by the 4.71 eV work function of BP.^[^ ^69^ ^]^ In Yang et al.’s report,^[^ ^50^ ^]^ HAuCl_4_ was reduced in situ on bare BPNs to homogeneous spherical Au NPs with an average size of 26 ± 4 nm. By the same method, Liu et al.’s group prepared BP–Au nanohybrids.^[^ ^70^ ^]^ According to Ouyang et al.’s report, Pt NPs with average sizes of around 4.2 nm were also added as artificial catalase onto BPNs through in situ reduction,^[^ ^51^ ^]^ the BPNs simultaneously acted as supporting substrates, reductants, and stabilizers during the formation of Pt@BP nanohybrids.

Chemical bonds such as covalent bonds, coordinate bonds, *π*‐bonding, and so on have also been adopted to construct BPNs‐based DDSs. For instance, Zhao et al. prepared NB@BPs using a method that involved combining Nile blue (NB) dye with BPNs via diazonium chemistry.^[^ ^65^ ^]^ In Fojtu et al.’s study, the d electrons of Pt (II) were donated to the vacant d orbitals of P from BPNs, and the as‐formed coordinate bonding between cisplatin, oxaliplatin, and BPNs gave sufficient stability to the DDS.^[^ ^47^ ^]^ A similar conclusion can also be obtained from Liu et al.’s report.^[^ ^71^ ^]^

### 2.2 Modified BPNs Platform

While bare BPNs seem promising for drug delivery, challenges remain: 1) susceptibility to aggregation and settling in real‐world use; 2) poor physiological stability and easy degradation in the presence of oxygen and water; 3) low drug‐loading efficiency via electrostatic interaction; and 4) lack of functional groups on their surfaces. Consequently, in real‐world applications, bare BPNs suffer from low drug loading, imprecise release, and suboptimal efficiency, leading some researchers to modify them. Among the many modifications addressing one or more of these problems, the main BP strategy has been electrostatic interaction between BPNs and modified components and the second approach uses a chemical reaction to conjugate BPNs.

To date, polyethylene glycol (PEG), PEI, polydopamine (PDA), poly(2‐ethyl‐2‐oxazoline) (PEOz), and human serum albumin (HSA) are common BPNs modification materials. These groups change electrical properties via –NH_2_ or cause reactions with other chemical bonds to introduce functionality or drugs. Amino compounds are by far the most common means of modifying BP, and four of these compounds have been used successfully:

1) PEG–NH_2_ has excellent biocompatibility and has been widely used in the field of biomedicine. BPNs modified with PEG–NH_2_ via electrostatic adsorption have exhibited good biocompatibility and physiological stability, with almost no aggregation or degradation, as observed through UV–vis absorption spectra and the Tyndall effect.^[^ ^44^, ^52^, ^53^, ^72^ ^]^ The abundant –NH_2_ groups transform the charge from negative to positive, enabling drug delivery. In Lv et al.’s report, BPNs–PEG–NH_2_ attracted poly(acrylic acid) (PAA)‐modified UCNPs by electrostatic force, and the as‐obtained multifunctional composite performed extremely well in PDT.^[^ ^52^ ^]^

**Figure 3:**
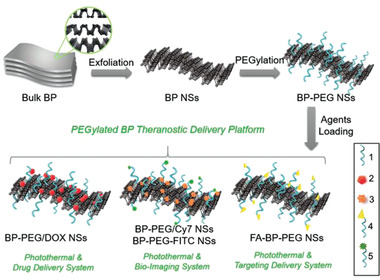
Schematic representation of the PEGylated BP theranostic delivery platform. 1) PEG–NH_2_ (surface modification), 2) DOX (therapeutic agents), 3) Cy7–NH_2_ (NIR imaging agents), 4) FA–PEG–NH_2_ (targeting agents), 5) FITC–PEG–NH_2_ (fluorescent imaging agents). Reproduced with permission.^[^ ^72^ ^]^ Copyright 2016, Wiley‐VCH.

As previously mentioned, in bare BPNs platforms, the electrical properties of drugs are changed by attaching PEI or another modifier to achieve loading onto the BP, but for drugs whose structure is difficult to change, another method is to alter the platform charge. Amino groups can also react as functional groups. Interestingly, a report similar to Dibaba et al.’s described combining UCNPs–PAA with BPNs–PEG–NH_2_ but via covalent conjugation instead of between the carboxyl group of PAA and the amine group of PEG–NH_2_. The results showed that PEG–NH_2_ not only reduced the degradability of BPNs but also was an efficient functional site.^[^ ^53^ ^]^ Tao et al. assembled PEG–NH_2_ onto BPNs to improve the material's biocompatibility and physiological stability without affecting its photothermal effect. Alternatively, PEGylated BPNs have been loaded with a different functional agent, including DOX for chemotherapy, cyanine7 (Cy7) and fluorescein isothiocyanate (FITC) for bio‐imaging, and folic acid (FA) for targeted delivery (**Figure** **3**).^[^ ^72^ ^]^

**Figure 4:**
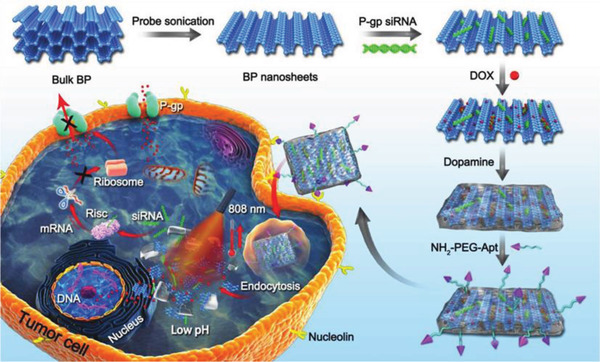
Schematic illustration of the procedure used to fabricate nanostructures and the combined chemo/gene/photothermal targeted therapy of tumor cells. Reproduced with permission.^[^ ^74^ ^]^ Copyright 2018, Wiley‐VCH.

2) PEI, like PEG–NH_2_, has abundant –NH_2_ groups, making it able to neutralize almost any negatively charged substance. In 2018, Chen et al.^[^ ^55^ ^]^ and Wang et al.^[^ ^57^ ^]^ prepared BP with PEI so that it could adsorb and protect siRNA from enzymatic degradation. BP was also modified with PEI by Zhang for integration with negatively charged gold nanoparticles.^[^ ^73^ ^]^ Since PEI has such abundant amino groups and is easily combined with BP, it is widely used to change the electrical properties of the substrate in a BP‐based drug delivery system.
3) PDA is a well‐known biomimetic polymer with high adhesive capacity that is easily synthesized by the self‐polymerization of dopamine in an alkaline environment. In BP‐based DDSs, PDA coating also confers enhanced stability and photothermal effects (**Figure** **4**),^[^ ^46^, ^74^ ^]^ and the –NH_2_ groups on the surface of PDA are able to covalently link with functional groups, such as via the carbodiimide reaction with the carboxyl group from chlorine6 (Ce6) and triphenyl phosphonium (TPP), and the reaction with boronic acid active sites in BTZ.^[^ ^46^, ^75^ ^]^
4) PEOz has been verified as a high‐molecular‐weight, long‐chain polymer with high water solubility, flexibility, and biocompatibility that has been approved by the United States Food and Drug Administration. Its unique charge‐reverse capability via the ionization of tertiary amide groups along the PEOz chain in low pH makes it an ideal pH‐responsive drug release modification for DDSs. Gao et al.’s report indicated that the unique tertiary amide groups in the main chain of PEOz gave PEOz‐coated materials more flexibility in physical and tumor microenvironments. Due to the similarity between the p*K*a value of PEOz and physiological pH, when p*K*a decreases, PEOz reverses from negative to positive charge through ionization of the tertiary amide groups, resulting in a PEOz‐modified, BP‐based DDS enriched in tumor site, availed endocytosis, and pH‐responsive drug release, leading to greater antitumor efficiency.^[^ ^46^ ^]^

The four amino‐modification strategies described above are the main ways to improve DDSs based on BP. Not only do they enhance the stability of BP, overcoming its greatest defect, but they also change its electrical properties and increase the number of functional groups, thereby boosting the efficiency and application potential of these DDSs.

An alternative to the amino‐modification strategy is HSA modification.^[^ ^45^ ^]^ It should be emphasized that HSA is used in the exfoliation of bulk BP, providing a more environmentally friendly method than traditional exfoliation processes. The prepared BP–HSA exhibits excellent biodegradability and biocompatibility, and it can act as a carrier for further loading of PTX via hydrophobic interactions. In this system, HSA can play a triple role: as an exfoliating and capping agent for BP nanosheets, and as a carrier of PTX.

In sum, each modification has its own advantages: the stronger hydrophilicity of PEG, which has longer circulation time in vivo; the stronger electropositivity of PEI; the greater adhesiveness of PDA, and so on. All of these modifications enable the construction of DDSs with more applications. Compared with pure BP substrate, the most important difference is that the modified substrate can ensure stability and improve biocompatibility in a physiological environment, and it can be loaded with drugs more effectively.

### 2.3 BPQDs Platform

Besides 2D BPNs, ultrasmall BPQDs and their derivatives also have become very popular. Thanks to their high on/off current ratio and flash memory effect,^[^ ^10^, ^76^, ^77^, ^78^, ^79^ ^]^ along with their excellent extinction coefficient,^[^ ^35^, ^80^ ^]^ wide spectral range from the visible to the near and mid‐infrared regions,^[^ ^35^, ^77^ ^]^ and high photothermal conversion efficiency,^[^ ^35^ ^]^ BPQDs are widely applied in electronics, as photothermal reagents, in photocatalysis, and in other areas. However, due to their small size and the challenges of constructing them, little research has been done on BPQDs in biomedical applications, especially in the field of drug delivery for anticancer treatments.

In what research has been done, the construction of these DDSs can be divided into two categories: modifying the BPQDs before they are loaded with the drug, and developing a hole structure, such as nanocores surrounded by a membrane camouflage constructed with BPQDs. Both strategies depend on the abundant phosphate groups on the surface of BP, which interact with drugs via hydrogen bonds, electrostatic forces, or covalent bonds.

**Figure 5:**
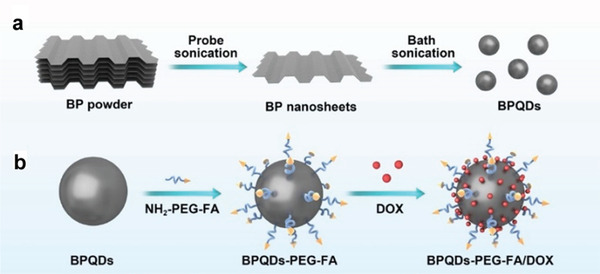
Schematic depiction of preparing (BPQDs)–PEG–FA/DOX. a) Schematic illustration of the preparation of BPQDs. b) Schematic illustration of BPQD‐based drug delivery system. Reproduced with permission.^[^ ^81^ ^]^ Copyright 2019, Molecular Diversity Preservation International.

When BPQDs bind to positively charged components through electrostatic interactions, the resulting changes in electrical properties improve biocompatibility, increase the number of functional groups, and confer targeted functionality, and the nanocarrier can then be used for drug delivery.^[^ ^80^ ^]^ For example, in 2017, BPQD nanocarriers coated with polyelectrolyte polymers (PAH) were used to deliver siRNA in human pluripotent teratoma PA‐1 cells. The apparent changes in zeta potential—from −39.02 ± 0.43 mV in bare BPQDs to +33.41 ± 1.05 mV for PAH–BPQDs and then −28.31 ± 1.38 mV after loading with siRNA—and the changes of size demonstrated the success of construction and loading.^[^ ^54^ ^]^ In 2019, Luo et al. developed a photo‐ and pH‐sensitive nanoparticle based on BPQDs for targeted chemo‐photothermal combination cancer therapy.^[^ ^81^ ^]^ In this work, NH_2_–PEG–FA was modified for targeting via electrostatic forces and was loaded with DOX utilizing chemical bonding with BPQDs through P—O—C bonds (**Figure** **5**). Lan et al. used a more sophisticated strategy to construct a BPQD‐based hybridized DDS.^[^ ^59^ ^]^ They first created a self‐assembling BPQD‐hybridized mesoporous silica framework via electrostatic interaction; this was followed by the in situ reduction of Pt nanoparticles as self‐supply oxygen on BPQD‐based hybridized DDS for enhancing PDT effect, then the use of “TLS11a” as a DNA aptamer to further target hepatocellular carcinoma decorated at BPQDs through Michael addition.

**Figure 6:**
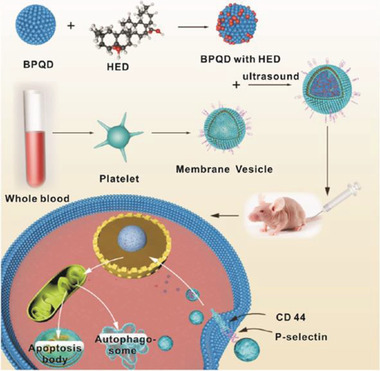
Schematic diagram of PLT@BPQDs–HED construction and targeted therapeutic mechanisms. Reproduced with permission.^[^ ^82^ ^]^ Copyright 2019, American Chemical Society.

Rather than loading directly on the surface of BPQDs without any encapsulation, the second approach is to introduce a capsule structure using a biofilm on the outside of the BPQDs, which acts to encapsulate them and prevent leakage, trapping the drug and BPQDs together within the membrane. Because quantum dots are so small, they can easily be encapsulated in a biofilm to form a camouflage. The drugs are then loaded onto the BPQDs and placed as nanocores inside nanoshells made of membranes. In 2019, Gui's group published two works on DDSs with membrane‐camouflaged BPQDs, which used red blood cell (RBC) membranes and platelet membranes (**Figure** **6**), respectively.^[^ ^48^, ^82^ ^]^ The drug loading capacities of these nanocomposites were 96.3% for DOX, 42.0% for KIR, and 89.5% for hederagenin (HED). This large capsule structure has three advantages: it provides a large space, which increases the drug load; it prevents BP degradation and uncontrolled loss of drugs; and it boosts drug biocompatibility, as the biofilm reduces the chance that the body will reject the drug. This unique way of constructing DDSs is only feasible with BPQDs.

Compared with nanosheets, the smaller size and higher specific surface area of a BPQDs platform can allow better absorption by the body, free travel within blood vessels, and enhanced permeability and retention (EPR) for targeted delivery. However, the BPQDs system has several disadvantages: it is difficult to operate, medicine delivery is challenging, and the body is apt to discharge BPQDs because they are so small.

### 2.4 BPHs Platform

In addition to BPNs and BPQDs, BP can also be constituents in hydrogel nanostructures for cancer therapy. To date, there has been little research on BP hydrogels, and nearly all of it is concentrated in the field of biomedicine. Hydrogels are considered excellent biological materials because of their special structure, high water content, minimal invasiveness, and biocompatibility.^[^ ^83^ ^]^ Phase transition in thermosensitive hydrogels is very flexible and easily reversed by external conditions, increasing the materials’ potential for use in controlled drug release.^[^ ^84^ ^]^ Given that hydrogels are recognized as remarkable drug delivery and controlled drug release platforms, combining them with the well‐known photothermal transduction capability of BP has led to their use in antibacterial, antiinflammatory, and anticancer applications.^[^ ^52^, ^81^ ^]^ As phosphorus is concentrated in human bones and has a unique ability to bind calcium ions, BP is an excellent promoter of biomineralization and bone regeneration.^[^ ^85^, ^86^, ^87^, ^88^ ^]^ Thus, BP‐based hydrogels have been successfully applied in water disinfection,^[^ ^89^ ^]^ rheumatoid arthritis treatment,^[^ ^90^ ^]^ bone regeneration,^[^ ^86^, ^91^ ^]^ and antitumor therapy.^[^ ^42^, ^43^, ^92^, ^93^ ^]^

**Figure 7:**
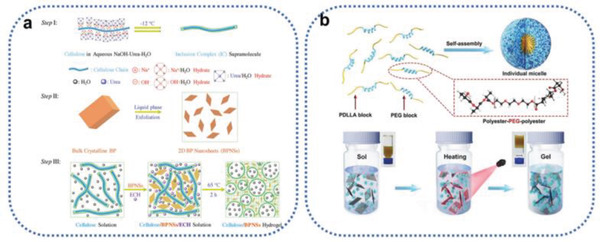
a) Synthesis route of cellulose/BPNS composite hydrogels. Step I: Dissolution of cellulose with the aid of NaOH, urea, and H_2_O at a low temperature of −12 °C. Step II: Exfoliation of BPNSs by means of liquid‐phase exfoliation. Step III: Fabrication of BPNS‐integrated cellulose hydrogels. Reproduced with permission.^[^ ^42^ ^]^ Copyright 2018, Wiley‐VCH. b) Schematic diagram of self‐assembly of PLEL into micelles and the thermogelation process of the BP@PLEL hydrogel induced by NIR irradiation. Reproduced with permission.^[^ ^43^ ^]^ Copyright 2018, Wiley‐VCH.

With the goal of constructing BPHs DDSs for cancer treatment, Xing et al.’s and Shao et al.’s groups reported two studies, on BP/cellulose and BP/thermosensitive (PLEL) hydrogels, respectively.^[^ ^42^, ^43^ ^]^ As seen in **Figure** **7**a, the BP/cellulose hydrogels were generated by heating to 65 °C of a solution of cellulose supramolecular chains, BPNs, and epichlorohydrin (ECH) as a crosslinker. Physical crosslinking and self‐aggregation of cellulose chains and chemical crosslinking via reactions between the –OH (cellulose) and epoxy groups (ECH) created BPHs. Figure 7b shows two polymeric chains of PLEL that can self‐assemble into core–shell‐like micelles in an aqueous solution. BP acts as a bridge for the micelles and forms a physically crosslinked gel structure that is affected by increasing temperature, triggered by the PTT of BPNs under NIR radiation. Both of the constructed hydrogels used the BP photothermal effect against tumors and provided insights into the construction of BPHs, but the studies did not explore the materials’ drug delivery capacity.

**Figure 8:**
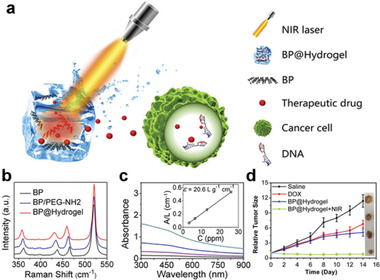
a) Schematic diagram of the working principle of BP@Hydrogel. BP@Hydrogel released the encapsulated chemotherapeutics under NIR‐light irradiation to broken the DNA chains, leading to the apoptosis induction. b) Raman spectra of BPNSs. c) Absorbance spectra of BPNSs dispersed in IPA at different concentrations. Inset shows the normalized absorbance at concentrations of 3.25, 6.5, 13, and 26, respectively. d) Corresponding growth curves of tumors in different groups of mice treated with PBS solution, DOX, BP@Hydrogel depot only, and BP@Hydrogel depot with laser irradiation. The relative tumor volumes were normalized to their initial size. Inset shows representative photographs of tumors removed from the killed nude mice. Reproduced with permission.^[^ ^92^ ^]^ Copyright 2018, PNAS.

In an analogous way, Qin et al. synthesized a drug‐encapsulated thermosensitive hydrogel.^[^ ^93^ ^]^ BPNs, pluronic F127 (a thermosensitive hydrogel matrix), and gemcitabine were added in sequence under stirring at 4 °C, and phase transition gradually occurred under NIR irradiation. The resulting DDS exhibited immense potential for tumor treatment. Qiu et al. constructed a BP@hydrogen DDS using agarose (**Figure** **8**a).^[^ ^92^ ^]^ The fabrication process involved mixing a melted agarose solution with PEGylated BPNs at 60 °C and loading DOX into the suspension, then rapidly cooling the mixture. A slight red shift was found after PEGylation and hydration with agarose due to adsorption, which partially hindered the oscillation of phosphorus atoms (Figure 8b). The extinction coefficient at 808 nm (Figure 8c, inset) indicates its greater penetration depth and potential application for in‐depth clinical treatment. As seen in Figure 8d, the final hydrogel with NIR showed high therapeutic efficacy in cancer treatment. It is worth noting that in the above methods, BP was encapsulated by the viscosity of the hydrogels. However, this viscosity weakens in vivo, so a more stable DDS that relies on chemical bonding is essential.

In 2018, Yang et al. fabricated a PDA‐modified, BPN‐containing poly(vinyl alcohol) (PVA) composite hydrogel, featuring crosslinked hydrogen bonding between the hydroxyl groups in the PDA and PVA chains.^[^ ^94^ ^]^ The addition of PDA was particularly critical, as it not only improved the biocompatibility and viscosity of the BPNs but also modified them into a building material for the hydrogel via solid bonding. This method can guide for the construction of BPHs. Drug loading with Congo red (CR) was performed by immersing the hydrogels in CR solutions. As the amount of BP increased, so did the drug load, because the hydrogel with BP had a greater crosslinking density and swelling ratio, and the functional groups (–NH– and −OH) on BP facilitated a higher drug load. Regrettably, this type of platform has not yet been further explored for cancer treatment.

## 3 Stimuli‐Responsive BP‐Based DDSs for Controlled Drug Release

To date, traditional DDSs through simple physical adsorption on nanostructures has led to irregular and immediate release after administration. The main drawbacks of these DDSs include the following: 1) drugs are released in an initial burst when the DDS first enters the physiological environment; 2) exposure of the drugs in vivo before they reach the target cells leads them to decompose or denature; 3) the drugs’ biodistribution can be altered by nonspecific cells and physiological conditions; 4) some drug molecules cannot distinguish between diseased and healthy cells, which can lead to collateral damage and undesired side effects for normal tissues and organs.^[^ ^85^, ^86^, ^87^, ^88^, ^89^, ^90^, ^91^, ^92^, ^93^, ^94^, ^95^, ^96^, ^97^ ^]^ Uncontrolled release increases drugs’ side‐effects and decreases their treatment efficacy, so to ensure precision, antitumor DDSs must be systematically managed to achieve high dosage as well as efficient and continuous treatment. This means that the ideal DDS should efficiently encapsulate high loads of anticancer drugs and not release them until the target cells are reached. Clearly, there is a need to design smart, stimuli‐responsive DDSs that will only release their loads at the desired time and location and in the appropriate amount, closing and opening at will. Smart DDSs would minimize off‐target effects in the physiological environment and maximize the payload in tumor treatment.

Tremendous effort has been directed at designing and fabricating stimuli‐responsive DDSs for controlled drug release. Benefit from the pathological lesions and different extracellular and intracellular environments between tumor and normal cell, such as blood vessels, elasticity, pH, reactive oxygen species (ROS), etc., the design of smart stimuli‐responsive DDSs for controlled drug release is motivated.^[^ ^98^, ^99^, ^100^, ^101^, ^102^ ^]^ These novel DDSs can be tailored to respond to pH, redox potential, enzymatic activation, thermal fields, magnetic fields, light, and ultrasound. Some are even responsive to combinations of two or more different stimuli.

To date, the research on BP‐based stimuli‐responsive DDSs is still at an early stage. With their unique characteristics, such as high photothermal conversion efficiency, good biocompatibility, and high biodegradability, they basically divide into those that respond to pH, light and GSH. In the following sections, we describe the state‐of‐the‐art developments in BP‐based stimuli‐responsive DDSs that are summarized in **Table** **2**, and a comprehensive perspective is presented for classifying their responsiveness to three stimuli, the combinations of dual stimuli will not be repeated illustrate.

**Table 2:** Classification of stimuli‐responsive BP‐based DDSs for controlled drug release

Stimuli‐responsive types	Manners of affect		Remarks	Ref.
pH	Protonation of drugs		Subtle pH changes in different sites of the human body are advantageous for the use of stimuli‐responsive DDSs. The manner by which pH affects the drug‐release behavior of BP‐based DDSs falls into four categories: drug protonation; BP degradation; coating or capsule decomposition; and destruction of the BP–drug bond. But individual differences between patients often affect drug release efficiency.	^[^ ^50^, ^66^, ^67^, ^68^, ^72^, ^74^, ^81^ ^]^
	Degradation of BP			^[^ ^55^, ^56^, ^82^ ^]^
	Decomposition of coating or capsule			^[^ ^46^, ^55^, ^66^, ^67^, ^74^ ^]^
	Destruction of bonding			^[^ ^46^, ^71^ ^]^
Near‐infrared region	Photothermal Therapy	Decomposition of coating and BP	Light irradiation has attracted a significant amount of attention as a noninvasive tool for remote spatiotemporal control of drug payload release at the desired site and time. Because its wavelength and intensity can be precisely tuned, the exposure duration and tissue location can be controlled. However, the penetrable ability and penetrable depth of light have the potential to result in a weaker release of the sample at deeper sites.	^[^ ^46^, ^68^, ^74^ ^]^
		Weaken the interactions		^[^ ^67^, ^68^ ^]^
		Destructed hydrogels		^[^ ^92^, ^93^, ^94^ ^]^
		Accelerated the movements of drugs		^[^ ^71^ ^]^
	Photodynamic Therapy	Degradation of BP by ROS		^[^ ^55^ ^]^
Glutathione	Reduction of disulfide bonds		Large amounts of GSH trigger a series of redox reactions, which enable DDSs to release drugs precisely in response to stimulation at tumor sites. Similarly, individual differences often lead to uncertainty about the effectiveness of treatment.	^[^ ^116^, ^117^ ^]^

### 3.1 pH‐Responsive DDSs

Among the various BP‐based stimuli‐responsive DDSs, those responsive to pH have received the most attention. Subtle pH changes in different sites of the human body are advantageous for the use of stimuli‐responsive DDSs. In the gastrointestinal tract, pH varies from the stomach (1.0–3.0), to the small intestine (6.5–7.0), to the colon (7.0–8.0).^[^ ^103^ ^]^ Notably, the extracellular pH of cancer cells tends to be significantly more acidic (pH = 6.5) than in tissues and blood (pH = 7.5).^[^ ^102^ ^]^ Considerable pH gradients exist within healthy cells; for example, lysosomes (4.5–5), endosomes (5.5–6), the Golgi apparatus (6.4), and the cytosol (7.4).^[^ ^97^ ^]^ These widely varying pH conditions in diverse biological systems have motivated the design of pH‐responsive DDSs. The manner by which pH affects the drug­release behavior of BP‐based DDSs falls into four categories: drug protonation of drugs; BP degradation; coating or capsule decomposition; and destruction of the BP–drug bond.

**Figure 9:**
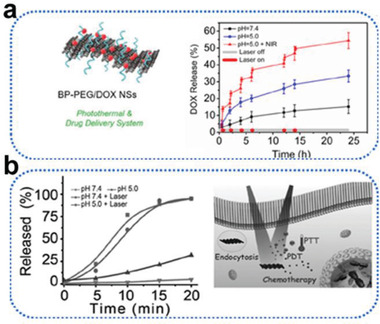
a) Schematic diagram and drug release kinetics of BP–PEG/DOX NSs at pH = 7.4 and pH = 5.0 (in the absence or presence of 1.0 W cm^−2^ NIR laser). Reproduced with permission.^[^ ^72^ ^]^ Copyright 2016, Wiley‐VCH. b) Schematic diagram and DOX released from BP‐DOX at pH 5.0 and 7.4 with or without 808 nm irradiation (1 W cm^−2^). Reproduced with permission.^[^ ^41^ ^]^ Copyright 2016, Wiley‐VCH.

The insolubility of some anticancer drugs, such as DOX, often impedes their effectiveness, but pH‐responsive DDSs can overcome this drawback through protonation of the drug via NH_2_. In the case of DOX, the protonation of the amino group on the drug's sugar moiety accelerates its solubility and facilitates its release in lower pH environments. In 2017, Tao et al. constructed a BP‐PEG platform as a robust DDS for loading DOX.^[^ ^72^ ^]^ As seen in **Figure** **9**a, via protonation of the amino group on DOX, the as‐prepared DDS achieved more DOX release at pH 5.0 over a span of 24 h (≈33.4%), while only ≈15.2% was released at pH 7.4. In the same year, Chen et al. studied DOX release behavior at pH 7.4 and 5.0.^[^ ^41^ ^]^ The release rate was six times greater at 5.0 than at 7.4, a finding ascribable to the accelerated solubility of DOX at pH 5.0 (Figure 9b). In 2019, Luo et al. reached a similar conclusion: only ≈16.4% release occurred at pH 7.4, compared with ≈37.8% at 5.0.^[^ ^81^ ^]^ The pH‐sensitive release of the chemotherapy drug MTX has also been studied; at a pH of 5.0, the release rate increased by about 1.25 times compared with at 7.4, due to the protonation of the amino group in the MTX molecules in the acidic environment.^[^ ^68^ ^]^ Analogous results have also been reported in other studies.^[^ ^66^, ^67^, ^74^ ^]^ These pH‐triggered DDSs demonstrate increased drug solubility, enhanced cellular uptake, and highly efficient drug delivery.

The degradation of BP in acidic environments is another major mechanism for controlling drug release. BP will degrade gradually in an acidic environment, and the resulting phosphate ions will in turn further increase the acidity, promoting drug release with BP‐based DDSs in a weakly acidic environment. Since the tumor environment is weakly acidic, this pH‐responsive drug release profile is favorable for tumor therapy. Chen et al. treated BP with PBS at pH 7.4 and 5.0, atomic force microscopy and dynamic light scattering observed a decrease size of nanosheets and phosphorus content in pH 5.0 PBS increased, indicating that PPBP nanosheets modified with PEG and PEI might have the potential for specific degradation in acidic lysosomes.^[^ ^55^ ^]^ They then extensively explored the lysosome escape and siRNA release mechanisms of PPBP‐siRNA using two fluorescent signals, and the results suggested that PPBP‐siRNA could lodge in endosomes or early lysosomes (pH = 5.0), then escape from these into the cytoplasm for use in gene therapy. The escape/release of siRNA from endosomes/lysosomes was attributable to the acidic environment; PPBP gradually degrade at an acidic pH, generating phosphate ions and strengthening the environment's acidity. The degradation products in turn increased the osmotic pressure and endosome swelling, facilitating siRNA release from the endosomes to the cytoplasm.^[^ ^104^, ^105^ ^]^

**Figure 10:**
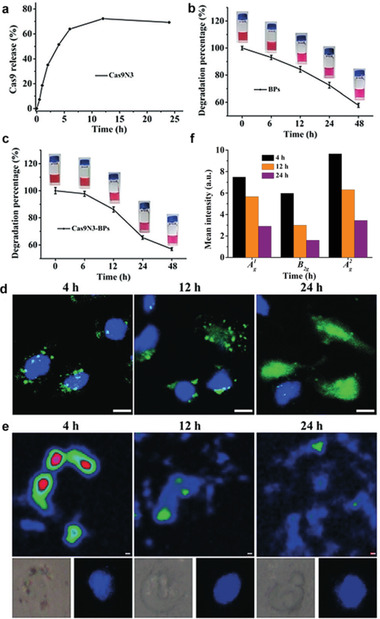
a) Percentages of active Cas9N3–sgRNA complexes released from Cas9N3–BPs at different time points. Time‐dependent degradation of b) bare BPs and c) Cas9N3–BPs in 10% FBS DMEM. d) Confocal laser scanning fluorescence imaging of MCF‐7 cells treated with Cas9N3_A488_–BPs at different time intervals. Blue and green fluorescence images show nuclear staining with DAPI and Alexa‐488, respectively (scale bar: 50 mm). e) Intracellular degradation of BPs in a selected cell monitored by Raman intensity mapping with the characteristic A_g_^1^ Raman peak of BP. Inset: the bright and nuclear (DAPI) image of a selected cell. The scale indicates 1.0 mm. f) Average intracellular Raman intensities of Cas9N3–BPs obtained from 120 × 120 points at different time intervals. Reproduced with permission.^[^ ^56^ ^]^ Copyright 2018, Wiley‐VCH.

Zhou et al. proved that the biodegradation of ultrathin BPNs can also trigger endosomal escape mechanisms.^[^ ^56^ ^]^ As seen in **Figure** **10**a, they observed the sustained release of Cas9N3 up to 72.2% in 12 h. During this time, the Cas9N3–BPNs degraded more slowly than bare BPNs (Figure 10b,c), indicating the drug's release was triggered by BPN degradation. They also studied cytosolic release and degradation using fluorescence (Figure 10d) and monitoring the Raman intensity mapping with the characteristic A_g_^1^ peak of BP (Figure 10e,f). By comparing markers of efficient drug internalization with the intensities of three characteristic Raman peaks, they determined that release was associated with the biodegradation of BPNs. In a later study, platelet‐membrane‐camouflaged BPQDs‐based DDSs constructed by Shang et al. also demonstrated pH‐responsive drug control release.^[^ ^82^ ^]^ In this situation, only 61.2 ± 5.2% of the HED was released by PLT@BPQDs‐HED at pH 7.4 within 48 h, compared with 95.4 ± 3.9% at pH 5.4. The degradation of BP in acidic conditions evidently promotes drug release that is favorable for tumor therapy. Overall, the good biodegradability BP‐based DDSs not only endows the system with good biocompatibility but also facilitates stimuli‐responsive drug release, suggesting the potential for using BP in drug delivery.

Decomposition of a coating or capsule can also depend on pH. In the construction of BP‐based DDSs, it is often necessary to introduce a coating to modify the BP so it will combine with a drug, or use a capsule to improve the platform's drug‐loading capacity and reduce irregular release. As a DDS enters the body or cancer cells, changes in pH tend to destroy its structure, causing materials to dissociate and peel away, which in turn leads to premature drug release. As described above, Chen et al. attributed the release of siRNA from an acidic endosome/lysosome to the degradation of BP.^[^ ^55^ ^]^ Meanwhile, the amino group of the PEI coating underwent protonation in the acidic environment, disturbing the electrostatic adsorption between PEI and BP, which led to further release of siRNA from the DDS.

PDA has been widely studied as a universal coating layer. A PDA “capsule” wrapped around drug‐loaded BP nanosheets has been shown to effectively suppress drug release bursts, and slow‐release anticancer drugs are favorable for extended, continuous tumor therapy.^[^ ^106^, ^107^ ^]^ More importantly, under acidic conditions, the pH sensitivity of the PDA coating induces its dissociation from the surface.^[^ ^66^, ^67^, ^74^ ^]^ Wu et al. examined drug release behaviors in PBS at different pH.^[^ ^67^ ^]^ Their results showed that the cumulative amount released was 11.2% at pH 7.4 but 17.7% at pH 6.8, and when the pH was 5.0, the cumulative amount reached 31.8%. Gao et al. reported 13.3% DOX release at pH 7.4 over 36 h, rising to 29.3% at pH 5.0 over the same time period.^[^ ^66^ ^]^ Hence, the PDA coating on the surface of BP acted as a protective capsule that controlled DOX release at the tumor site.

PEOz, another long‐chain polymer with unique tertiary amide groups in the main chain, has a similar p*K*a to physiological pH. At a pH lower than the material's p*K*a, the ionization of tertiary amide groups leads to charge reversal. The positive charges on the nitrogen atoms of PEOz main chains can result in electrostatic repulsion, which loosens the outer shell in the slightly acidic tumor cell microenvironment and accelerates the internal release of anticancer drugs. Gao et al. found that nearly 30% of DOX was released at pH 5.0, whereas only 11% was released at pH 7.4.^[^ ^46^ ^]^

Overall, under the acidic conditions of the tumor microenvironment, external coatings or capsules partially peel away from BP, resulting in faster drug release due to the destruction of bonds between the BP and the drugs. Controlled release can be achieved by building pH‐sensitive bonds. BTZ can be loaded onto the surface of a BP‐based nanoplatform through the reversible covalent bond between the boronic acid active site in BTZ and the catechol in PDA‐modified substrates. When pH drops, the pH sensitivity of the catechol‐BTZ bond contributes to significantly accelerated release.^[^ ^46^ ^]^ Liu et al. reported that only 16.1% of DACHPt was released at pH 7.4, whereas 35.4% was released at pH 5.0.^[^ ^71^ ^]^ At the higher pH, DACHPt was released from BP/DACHPt by the substitution of chloride ions instead of BP in PBS. In acidic conditions, accelerated release may be ascribed to the increased absorption of hydrogen ions on BP, which further weaken the coordination between DACHPt and BP. The advantages of this strategy are its simplicity and convenience, as system construction is a one‐pot process.

### 3.2 Light‐Responsive DDSs

Among the several stimuli exploited in smart DDSs, light irradiation has attracted a significant amount of attention as a noninvasive tool for remote spatiotemporal control of drug payload release at the desired site and time. Because its wavelength and intensity can be precisely tuned, the exposure duration and tissue location can be controlled, and photo‐regulated activation is regarded as noninvasive, light‐responsiveness is considered crucial to boost local effective drug accumulation while minimizing side effects, resulting in improved therapeutic outcomes.^[^ ^108^, ^109^, ^110^ ^]^ The ultraviolet (10–400 nm), visible, or NIR regions (650–900 nm) of the light spectrum can be used to trigger or stimulate drug release from appropriately designed nanocarriers. UV irradiation is much more cytotoxic than the other regions of the light spectrum, and its inability to penetrate deeply into tissue is another disadvantage. Thus, only wavelengths below 650 nm are considered suitable to trigger drug release for the topical treatment of pathological states affecting the skin and mucosa.^[^ ^111^ ^]^ However, NIR has better transmission through tissue due to its lower absorption and scattering in tissue (penetrating into the body about 10 cm) because it is minimally absorbed by hemoglobin, water, and lipids, and it causes less damage to cells than visible light due to its lower energy per photon.^[^ ^112^ ^]^

Hence, light‐responsive DDSs may provide practical methods for the remote‐controlled release of payload molecules using an NIR laser as the excitation source. In the BP‐based DDSs, PTT induced by NIR has become a label property of BP. Its distinguished PTT effect triggered by NIR offers an excellent choice for most light‐responsive DDSs. The temperature of BP‐based DDSs exposed to an NIR laser gradually increases, leading to a significant cumulative increase in the amount of drug released. To date, there have been numerous studies on drug release from stimuli‐responsive BP‐based DDSs induced by PTT. For example, Tao et al. and Luo et al. achieved higher drug release by utilizing on/off control of NIR.^[^ ^72^, ^81^ ^]^ Chen et al. and Ou et al. observed further facilitation of drug release (over 90%) by irradiation with NIR.^[^ ^41^, ^58^ ^]^

Some reports have sought to explain the specific mechanism of PTT‐induced drug release, and their findings fall into four broad categories: i) PTT induces decomposition; ii) PTT weakens the interactions between drug and nanocomposite; iii) PTT destroys BP hydrogels; and iv) PTT accelerates the movements of drugs. Each of these will be discussed below.

In Zeng et al.’s report, a PDA‐modified BP nanocapsule exhibited pH‐responsive drug‐release behavior, resulting in nearly 33.4% DOX release after treatment with an NIR laser (808 nm, 1.0 W cm^−2^, 6 min for each pulse); at pH 5.0, it reached 46.9% after four irradiations.^[^ ^74^ ^]^ To clarify the release mechanisms, the researchers examined the material's photothermal properties by increasing the solution temperature to 26.5 °C after NIR laser irradiation. Examination using transmission electron microscopy (TEM) after NIR irradiation showed that the PDA layer outside the BP had partially broken up and peeled off, and decomposition of the internal BP could be observed, indicating the degradation of the PDA film and gradual destruction of BP under NIR irradiation. They concluded that NIR‐triggered PTT led to the decomposition of the PDA coating and the BP platform, which promoted the drug's release. Gao et al.’s group provided the same explanation for their stimuli‐responsive DDS.^[^ ^46^ ^]^ Zhang et al. posited that under exposure to NIR, the movement of oxygen atoms in the aqueous solution was accelerated, resulting in the destruction of the composite structure and the gradual degradation of BP, which further induced drug release.^[^ ^68^ ^]^

Zhang's and Wu's groups studied the mechanisms for controlling drug delivery using mesoporous, silica‐coated, polydopamine‐functionalized reduced graphene oxide and graphene quantum dots capped with magnetic mesoporous silica nanoparticles,^[^ ^113^, ^114^ ^]^ finding that the heat generated from BP decreased the electrostatic forces between drug and nanocarrier.^[^ ^67^, ^68^ ^]^ Zhang et al. reported that after treatment with a 808 nm NIR laser, the amount of free MTX measured under these conditions was ≈1.5 times what occurred without laser irradiation. Wu et al. found that all release rates were promoted during laser irradiation. They then studied intracellular DOX release. The cell nucleus and DOX were labelled with blue and red fluorescence, respectively. Without NIR irradiation, only weak red fluorescence was observed, compared with intense intracellular red fluorescence after irradiation with a NIR laser. In addition, an NIR‐triggered heat effect accelerated the release of DOX, as shown by intracellular release. The interactions between the drugs and their nanoplatforms were attributed to electrostatic forces, which weakened as the temperature increased, resulting in the easy release of the drugs from the DDSs.

**Figure 11:**
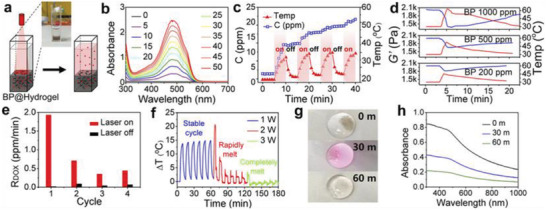
NIR‐light‐controlled BP@Hydrogel drug delivery platform. a) Schematic representation of the BP@Hydrogel drug delivery platform and the physical map shown in Inset. b) Absorbance spectra of released DOX. c) Photo‐controlled temperature increase and release of DOX from BP@Hydrogel depot. d) Rheological curves (blue line) and corresponding temperature curves (red line) of BP@Hydrogel with different BP concentrations under 1 W cm^−2^ NIR‐light irradiation. e) Release rate of DOX with and without laser exposure. f) Temperature change versus time under 808 nm laser with different powers. g) BP@Hydrogel under different laser exposures. h) Absorbance spectra of DOX under 808 nm laser exposure. Reproduced with permission.^[^ ^92^ ^]^ Copyright 2018, PNAS.

As we have already described, some BP‐based DDSs constructed in hydrogel systems can greatly improve biocompatibility and drug loading. Most importantly, a hydrogel system is particularly helpful for controlled drug release, especially in PTT‐stimulated release. Because the crosslinking force between hydrogels is temperature sensitive, the phase transition reaction responds to changes in temperature, making this reaction very useful for controlling drug release. Due to the excellent photothermal conversion efficiency of BP, most BP–hydrogel DDSs exhibit NIR‐responsive release. Qiu et al. employed 1 W cm^−2^ 808 nm NIR to irradiate a BP@low‐melting‐point agarose hydrogel for 5 min “ON,” following this with turning it “OFF” for 5 min (**Figure** **11**).^[^ ^92^ ^]^ Using UV to conduct real‐time monitoring of the amount of drug released, and a thermocouple to detect temperature changes, they found that as the NIR irradiation time increased, the temperature and the amount of DOX released rose dramatically, and the increase amplitude unchanged in the “OFF” phase, indicating the BP@hydrogel operated as an effective optical switch for drug release. The BP converted light to thermal energy, raising the temperature in the hydrogel matrix. The agarose hydrogel then underwent reversible hydrolysis and softening, which accelerated the diffusion of the drug from the matrix to the environment. They further assessed the biodegradation of BP@hydrogel under different laser powers, finding that it proceeded well under low laser power as the temperature increased by more than 10 °C. When the laser power was increased to 1.5 W, the temperature increased dramatically, the hydrogel became molten, and the BP and DOX diffused throughout the solution. Under 2 W of irradiation, the BP@hydrogel melted completely, resulting in polymer degradation due to hydrolysis of the ester linkages into segments with reduced molecular weight, oligomers, and monomers, and finally into carbon dioxide and water. The effects of these three different irradiation powers on BP@hydrogel proved that low‐power irradiation was conducive to drug release, and that the hydrogel and BP would gradually degrade when the power was increased, highlighting the great potential of this approach for clinical applications.

**Figure 12:**
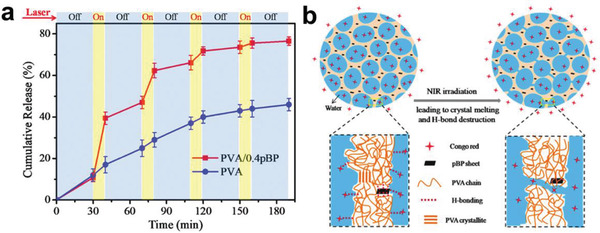
a) Cumulative release profile of CR from PVA hydrogels and PVA/0.4pBP hydrogels in response to periodic laser switching. b) Schematic representation of release of CR from PVA/pBP composite hydrogels with and without NIR irradiation. Reproduced with permission.^[^ ^94^ ^]^ Copyright 2018, The Royal Society of Chemistry.

Similarly, Qin et al. found that DEM release increased from 63.39% to 81.20% under NIR irradiation, caused when PTT of BP softened F127 thermo‐sensitive hydrogel and released the drug.^[^ ^93^ ^]^ Yang et al. found that pure PVA hydrogel was slow for drug release, exhibiting the characteristics of a Fickian release pattern, but PVA with BP added showed rapid drug release under NIR irradiation, slowing when the NIR was turned off.^[^ ^94^ ^]^ As seen in **Figure** **12**a, after the first cycle, 40% of the CR in BP@PVA hydrogel had been released, compared with only 15% from the pure PVA hydrogel after the first cycle. After four cycles, the BP@PVA hydrogel had released 78% of the drug, which was almost twice as much as with pure PVA. The increased release amount and highly controllable release rate under NIR irradiation can be explained by the photothermal response of microstructures in hydrogels (Figure 12b). Under NIR irradiation, PTT causes the hydrogel to heat up and then melt, which breaks the original 3D physically crosslinked networks (PVA crystallites and hydrogen bonding between PVA chains) and the hydrogen bonding between CR and the hydrogel. When the NIR is turned off and the hydrogel gradually cools down to room temperature, the bonds reform, reconstructing the hydrogel and thereby once again limiting the drug's release. This accounts for the slow‐release property of BP@PVA hydrogel and makes it an ideal DDS.

In light of previous studies, Liu et al. determined that brief NIR laser irradiation (10 min) was able to induce momentary burst releases.^[^ ^71^ ^]^ They then explored the stability of a DDS with anticancer drugs to ensure these could still exert antitumor effects under NIR‐induced release, and they found that the molecular weights after NIR irradiation were the same as in the original solution. This burst release occurred due to hyperthermia under NIR, which accelerated drug movement and facilitated its release. This mechanism may be present in all BP‐based DDSs used for PTT‐induced drug release.

Light‐triggered PDT of BP has also opened up the possibility of releasing drugs on demand. Chen et al. have proposed that exposure to light at 660 nm will trigger the production of ROS when BPs are subjected to PDT; a large amount of ROS leads to degradation of the BP substrate, which triggers drug release.^[^ ^55^ ^]^ But to date, little research has been done on light‐triggered PDT for controlled drug release.

### 3.3 GSH‐Responsive DDSs

GSH‐responsive DDSs often depend on redox. Cancerous cells differ from normal cells in many ways, one of which is manifested by excess GSH levels.^[^ ^115^ ^]^ Large amounts of GSH trigger a series of redox reactions, enabling DDSs to release drugs precisely in response to stimulation at tumor sites. Generally, GSH disrupts the interactions between DDSs and leads to disassembly; in particular, intracellular GSH may trigger the reduction of disulfide bonds in polymeric assemblies. Chan et al. assembled PLGA and PEI via disulfide bonds, then observed that the disulfide bonds were reduced to sulfhydryl with 10 × 10^−6^ m GSH, resulting in a particle size reduction from 2851 to 139 nm that was confirmed by ^1^H NMR, Raman, FTIR, and XPS spectra.^[^ ^116^ ^]^ The bioresponsive release rate increased from 17.9% in PBS to 45.1% with 10 × 10^−6^ m GSH. Zhang et al. incorporated DOX inside DDSs, using BPQDs as the cap.^[^ ^117^ ^]^ When the concentration of GSH increased, the disulfide bonds broke, which disrupted the integrity of the nanocarrier and removed the BPQD cap, allowing the release of the DOX. The drug release ratio gradually increased with the amount of GSH, reaching 64.8% DOX released within 12 h in the presence of 10 mm GSH; this was confirmed by the enhanced characteristic absorption peak of DOX.

## 4 BP‐Based DDSs for Cancer Therapy Applications

Despite prodigious advances in our understanding of the molecular, cellular, and physiological mechanisms involved in the initiation and progression of cancer, it remains one of the leading causes of mortality across all age groups. To date, tremendous effort has been poured into developing therapeutic approaches to overcome tumor invasion and metastasis. Current cancer therapies—chemotherapy, PTT, PDT, RT, immunotherapy, and so on—are clinically successful as either monomodal or multimodal approaches. So far, various types of nanomaterials have been developed as drug carriers for the complete inhibition of tumors, including gold nanoparticles,^[^ ^118^, ^119^ ^]^ bismuth‐based nanoparticles,^[^ ^120^ ^]^ calcium‐based biomaterials,^[^ ^121^ ^]^ and carbon nanomaterials.^[^ ^122^, ^123^ ^]^ The potential of BP‐based DDSs for therapeutic cancer applications, including chemotherapy, PTT, GT, PDT, and combination therapy, has been extensively investigated in the recent past, but there is a long way to go before discoveries will translate into clinical applications. Cancer imaging has become a hotspot in tumor research, generating a new need for integrated diagnosis and treatment using antitumor DDSs. In this section, we describe BP‐based DDSs that have demonstrated value in cancer treatment through mono‐, bi‐, and/or multimodal therapies; these are summarized in **Table** **3**. We also discuss the integrated application of imaging‐guided therapy.

**Table 3:** Summary of the characteristics of the major types of cancer therapies

Classification	Types	Materials	Remarks	Ref.
Monotherapy	Chemotherapy	BP–OP	Chemotherapy has achieved considerable success but is hindered by limited treatment efficacy and undesirable side effects, and it can lead to drug resistance.	^[^ ^47^ ^]^
	PTT	BP–Au	PTT has received increased attention due to its noninvasiveness, biocompatibility, and precision targeting of tumors, but it fails to kill metastatic tumor cells.	^[^ ^50^ ^]^
		NB@BP		^[^ ^65^ ^]^
	PDT	UCNPs−BPNs	PDT has become a promising treatment modality due to its significant effectiveness, specific spatiotemporal selectivity, minimal invasiveness, and limited side effects, but it is limited by the excitation wavelength and hypoxia at the tumor site.	^[^ ^52^ ^]^
		Pt@BP		^[^ ^51^ ^]^
		Cy5–dHeme–BPNS–FA		^[^ ^195^ ^]^
		R–MnO_2_–FBP		^[^ ^196^ ^]^
	GT	Cas9N3–BPs	An ideal DDS for GT is the key to expanding its practical application, to protect oligonucleotides from enzymatic degradation, promote cell uptake with high transfection efficiency, and intelligently release oligonucleotides from the DDS.	^[^ ^56^ ^]^
	SDT	Au@BP	SDT as a new cancer therapy with unique advantages in tissue permeability has emerged gradually in recent years but is often limited by poor stability, toxicity, biodegradability, and low ROS production yield.	^[^ ^217^ ^]^
Bimodal therapy	PTT/chemotherapy	BP–DOX@PDA–PEOz	PTT enhances drug uptake, improves targeting, and prolongs drug release time for chemotherapy. Chemotherapy is a systemic treatment paradigm for killing metastatic tumor cells when PTT fails.	^[^ ^46^ ^]^
		BP–HSA–PTX		^[^ ^45^ ^]^
		BPQDs–PEG–FA/DOX		^[^ ^81^ ^]^
		BP–PEG–FA/DOX		^[^ ^72^ ^]^
		BP@MTX–HA		^[^ ^68^ ^]^
		BP–AuNPs		^[^ ^70^ ^]^
		BP@Hydrogel		^[^ ^92^ ^]^
		BP–GEM–GEL		^[^ ^93^ ^]^
	PTT/PDT	Genipin–polyglutamic acid–Fe_3_O_4_–CDs@BPQDs	PTT can promote the cellular uptake of photosensitizers and accelerate blood flow to increase vascular oxygen saturation, which can enhance PDT efficacy.	^[^ ^226^ ^]^
		UCNP–BPNS		^[^ ^53^ ^]^
		BPs@Au@Fe_3_O_4_		^[^ ^48^ ^]^
		BP@PEG/Ce6		^[^ ^44^ ^]^
		BP@PDA–Ce6&TPP		^[^ ^75^ ^]^
		BP–PEI/AuNPs		^[^ ^73^ ^]^
	PTT/GT	BPQDs@PAH/siRNA	By increasing the tumor cells’ uptake of genes and accelerating gene release from DDSs, PTT can cooperatively enhance GT and lead to more efficient gene delivery. GT can in turn enhance PTT by specifically inhibiting heat shock protein expression and reducing the resistance of cancer cells to heat damage.	^[^ ^54^ ^]^
		BP–PEI–siRNA		^[^ ^57^ ^]^
	PTT/CDT	BP@Cu	Without additional conditions such as light and ultrasound, only H_2_O_2_ can be used to eliminate tumors all the time, so it is often combined with PTT for collaborative antitumor action.	^[^ ^60^ ^]^
		dSIS–BPNs–PDA@Ag		^[^ ^238^ ^]^
Multimodal therapy	PTT/PDT/chemotherapy	BP–DOX	The local, continuous hyperthermia caused by PTT promotes the release of drugs from DDSs to enhance chemotherapy and simultaneously increase membrane permeability to promote PDT. Chemotherapy targets the nucleus, whereas PDT usually causes oxidative damage to the organelles. PTT provides more opportunities for chemotherapy and PDT to get inside cancer cells.	^[^ ^41^ ^]^
		BPNs–PDA–PEG–PEITC/DOX		^[^ ^67^ ^]^
	PTT/PDT/GT	PPBP–siRNA	An ideal carrier and a combination of PDT/PTT can address the deficiencies of GT. BP not only can be used as a high‐quality DDS for GT but also is a very efficient photosensitizer for PDT and possesses NIR photothermal properties for PTT.	^[^ ^55^ ^]^
	PTT/chemotherapy/GT	BP–R–D@PDA–PEG–Apt	By the co‐encapsulation of a drug and siRNA into a photothermal nanocarrier, the combination can be realized within a single nanostructure. PTT can enhance the tumor cell uptake of the drug and siRNA, resulting in a remarkable trimodal synergistic therapeutic effect.	^[^ ^74^ ^]^
	PTT/PDT/CDT	FeOCl@PB@PDA@BPQDs@Mn	Not only can PTT promote cellular uptake and accelerate blood flow to increase the vascular oxygen saturation of PDT, but it also is appropriate for CDT, enhancing the efficacy of both PDT and CDT.	^[^ ^115^ ^]^
	PTT/PDT/chemotherapy/immunotherapy	BP–DcF@sPL	Immunotherapy can distinguish between cancer cells and normal cells, thereby effectively improving treatment efficiency and minimizing side effects. PTT, PDT, and chemotherapy can also trigger the immune system.	^[^ ^58^ ^]^
	PTT/PDT/CDT/immunotherapy	FePt/BP–PEI–FA	Apart from reciprocal promotion among PTT, PDT, and CDT, PTT is capable of enhancing immunotherapy by inhibiting metastatic tumor growth, because it stimulates the host immune system to release tumor antigens into the tumor microenvironment and promotes the expression of tumor‐derived antigens to the T cells.	^[^ ^249^ ^]^
Other therapy	Chemotherapy/antiinflammatory therapy	RBC@BPQDs‐DOX/KIR	Infiltration of immune cells promotes tumorigenesis, invasion, and metastasis in tumor microenvironments. Antiinflammatory therapy is particularly necessary for removing tumors and preventing drug resistance.	^[^ ^48^ ^]^
	Cell autophagy and apoptosis	PLT@BPQDs‐HED	Autophagy protects organelles from damage while at the same time killing the tumor. Apoptosis is one mechanism of cell death. The promotion of mitochondria‐mediated cell apoptosis and autophagy is beneficial against tumors.	^[^ ^82^ ^]^

### 4.1 Monomodal Cancer Therapy

Only limited success has been achieved with current clinical treatment options due to the complexity, diversity, and heterogeneity of tumors. Some monotherapies, such as chemotherapy, RT, and high‐intensity focused ultrasound therapy, have been applied clinically to suppress tumor proliferation, with remarkable results.^[^ ^124^ ^]^ Other monotherapies, such as PDT, PTT, GT, and immunotherapy, although still in the preliminary stages of clinical investigation, have also demonstrated high anticancer efficacy in numerous laboratory and preclinical research studies, showing substantial promise for translation into clinical use. BP‐based DDSs are being widely investigated for therapeutic applications, including chemotherapy, PTT, PDT, GT, sonodynamic therapy (SDT), chemodynamic therapy (CDT), and immunotherapy due to their outstanding biocompatibility, large specific surface area, high quantum yield, and photothermal conversion efficiency.

#### 4.1.1 Chemotherapy

As a cancer‐treatment modality, chemotherapy has achieved considerable success in prolonging the lives of millions of patients and continues to be the first line of treatment for most cancers.^[^ ^125^, ^126^, ^127^ ^]^ However, numerous preclinical and clinical studies have shown that it is difficult to completely eradicate malignant tumors with a single chemotherapy, mainly due to limited treatment efficacy and undesirable side effects. Some conventional drugs, such as DOX, PTX, BTZ, and oxaliplatin, have been implemented in clinical chemotherapy, but the rapid clearance and nonspecific distribution of these chemotherapeutics severely diminish their effectiveness.^[^ ^128^ ^]^ Nonspecific drug accumulation also can result in overdose because safe dosages may not completely eradicate tumors, so patients inevitably suffer the adverse side effects arising from systemic toxicity.^[^ ^125^, ^129^ ^]^ In addition, since mutations favorable to the survival of tumor cells are selected as chemotherapy progresses, prolonged drug use often induces the intrinsic and acquired resistance pathways of cancer cells against chemotherapeutics.^[^ ^130^, ^131^ ^]^

To tackle these problems, the aim in designing DDSs has been to effectively target the tumor site, reduce the unintentional loss of drugs, enhance cellular uptake, and improve chemotherapeutic effects. An investigation of BP's potential to target cancer tissue with clinically utilized platinum agents was reported.^[^ ^47^ ^]^ A well‐characterized human ovarian cell line, A2780, derived from the tumor tissue of an untreated patient, was exposed to assess cytotoxicity. The researchers found that after the BP was loaded with oxaliplatin (designated as BPOP), the difference between BP and BPOP cell‐growth inhibition was more than 21%, indicating the enhancement of cancer growth suppression by oxaliplatin. Moreover, they observed that the effectiveness of oxaliplatin gradually increased with higher concentrations of BP, even when the concentration of oxaliplatin was kept the same, suggesting a synergic or potentiating effect of the nanocarrier and the drugs. This enhancement of anticancer activity was due to the enhanced cellular uptake of BP‐based DDS loaded with oxaliplatin.

#### 4.1.2 PTT

Among various anticancer treatments, NIR PTT mediated by BP‐based DDSs has received increased attention due to its noninvasiveness, biocompatibility, and precision targeting of tumors via the use of external laser irradiation with adjustable intensity, to minimize both damage to the surrounding healthy tissues and systemic cytotoxicity.^[^ ^132^, ^133^, ^134^, ^135^, ^136^, ^137^, ^138^ ^]^ As water and blood cells minimally absorb NIR, it can penetrate more deeply into cancer cells than UV/visible light. Photothermal conversion agents harvest the energy from light and transform it into local heat to increase the temperature of the surrounding environment; this heat can be used for PTT to achieve the thermal ablation of tumor cells and trigger cell death.^[^ ^139^, ^140^, ^141^, ^142^, ^143^, ^144^, ^145^, ^146^ ^]^

Ideally, photothermal agents should exhibit strong absorbance in the NIR region, excellent biocompatibility, high conversion efficiency, and low or no toxic side effects. So far, a large number of optically sensitive nanomaterials have shown great promise in PTT, including Au nanomaterials^[^ ^147^, ^148^, ^149^, ^150^ ^]^ and carbon nanomaterials.^[^ ^151^, ^152^, ^153^ ^]^ 2D nanomaterials with distinct advantages in photothermal transfer efficiency have also been reported, such as graphene oxide, transition metal dichalcogenides, and transition metals oxides, which have shown encouraging therapeutic efficacy in PTT studies of cancer cells in vitro and in vivo.^[^ ^154^, ^155^, ^156^, ^157^, ^158^ ^]^ However, most of them still suffer from poor biodegradability, and concerns remain about potential deleterious effects.^[^ ^6^ ^]^

**Figure 13:**
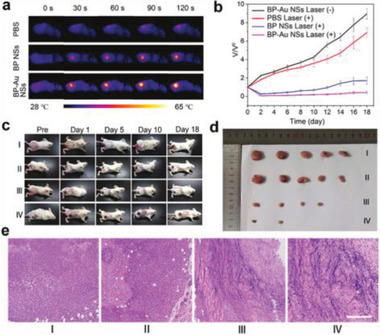
a) Infrared thermal images of 4T1 tumor‐bearing mice irradiated with an 808 nm laser (2 W cm^−2^) for 2 min. b) Tumor growth curves indifferent groups subjected to different treatments. Data are presented as mean ± SD. c) Photographs of the 4T1 tumor‐bearing mice after laser irradiation. d) Photographs of tumors collected from different groups of mice at the end of the 18 day treatment. e) Micrographs of H&E stained tumor tissue obtained from different groups (group I: PBS + laser; group II: BP–Au NSs; group III: BP NSs + laser; group IV: BP–Au NSs + laser; scale bar: 200 µm). Reproduced with permission.^[^ ^50^ ^]^ Copyright 2017, The Royal Society of Chemistry.

The excellent optical features and remarkable biodegradability of BP and BP‐based DDSs have stimulated an upsurge in the area of PTT.^[^ ^41^, ^159^ ^]^ In 2017, two reports on BP–Au and NB@BP demonstrated their potential in PTT for tumor treatment.^[^ ^50^, ^65^ ^]^ Yang et al.^[^ ^50^ ^]^ investigated the photothermal properties of BP–Au and bare BPNs, demonstrating that Au loading can enhance the photothermal capability of BP. They then examined in vitro PTT using an MTT assay of 4T1 cells irradiated with an 808 nm NIR laser (2 W cm^−2^); 75% of the cancer cells were destroyed after incubation with 30 µg mL^−1^ BP–Au, while more than 60% of the 4T1 cells were still alive in the BP NS group, indicating the 4T1 cells in the BP–Au group suffered more severe photothermal damage. in vivo photothermal therapy in which temperature change and tumor size are recorded, and photographs and H&E stained images are prepared, has proven that even bare BP has a certain photothermal effect, but BP–Au shows higher performance in photothermal tumor therapy (**Figure** **13**).

Similarly, the NB@BP constructed by Zhao et al.^[^ ^65^ ^]^ exhibited high efficiency as a PTT agent during in vitro and in vivo tumor ablation; ≈90% of the cells were killed in the presence of only 50 ppm of NB@BPs after exposure to the NIR laser in vitro, and tumors in mice treated with NB@BPs gradually shrank until they were completely gone within 16 days. BP modified with Nile blue fluorescent dye has also shown strong NIR fluorescence, making it usable in multifunctional nanomedicine for imaging‐guided photothermal cancer therapy.

#### 4.1.3 PDT

PDT has become a promising treatment modality and has been approved for clinical use, including to treat cancers of the lung, esophagus, and skin.^[^ ^160^, ^161^, ^162^, ^163^, ^164^, ^165^, ^166^, ^167^ ^]^ PDT offers significant effectiveness, specific spatiotemporal selectivity, minimal invasiveness, and limited side effects, making it an alternative for patients who are not candidates for radical operations.^[^ ^6^, ^160^, ^168^, ^169^, ^170^ ^]^ PDT has three major components: light, PS, and oxygen molecules. A specific light source of appropriate wavelength provides energy for activation. A PS administered beforehand and taken up by tumor cells harvests this light and engages in photodynamic reactions with oxygen‐containing substrates (e.g., molecular oxygen, water) to produce SO or ROS. This process induces selective damage to tumors by destroying their tissues and the vasculature surrounding them, killing cancer cells.^[^ ^124^, ^164^, ^170^, ^171^, ^172^, ^173^, ^174^, ^175^, ^176^ ^]^

Conventional organic photosensitizers currently in use exhibit poor water solubility, low stability, and low quantum yield, and other ambiguous security issues.^[^ ^177^, ^178^, ^179^ ^]^ Consequently, certain semiconductors and photocatalysts have been proposed as new PS agents.^[^ ^180^, ^181^ ^]^ BP is a metal‐free semiconductor with a high ^1^O_2_ quantum yield and thus could be a promising therapeutic agent for use in PDT.^[^ ^35^, ^36^, ^72^, ^159^, ^182^ ^]^ However, its excitation wavelength is in the visible light region, which has limited penetration depth, so tissue interference impedes BP's biomedical application in this capacity. Thus, it is often necessary to load it with molecules that can be activated in the NIR region. For example, Lv et al. integrated UCNPs with BPNs, enabling modification of the NIR irradiation to visible light to further stimulate the BPNs.^[^ ^52^ ^]^ The results indicated that 808 nm laser light caused the largest amount of ROS, and HeLa cells incubated with UCNPs–BPNs under irradiation at 808 nm exhibited a low viability of 29.8% in vitro. The antitumor performance in vivo was even stronger, clearly demonstrating the potential applicability of UCNPs−BPNs as a PDT antitumor agent with a single 808 nm laser.

As with most of the current PDT agents, another disadvantage that limited BP's in vivo application is the complex hypoxic microenvironment of tumors. This curbs the generation of ROS by PS, and the rapid local depletion of oxygen further aggravates tumor hypoxia, leading to even lower therapeutic efficacy. Worse still, hypoxia results in the accumulation of H_2_O_2_, which activates hypoxia‐inducible factors related to signaling pathways, generating therapy resistance.^[^ ^183^, ^184^, ^185^, ^186^, ^187^, ^188^, ^189^, ^190^, ^191^, ^192^, ^193^, ^194^ ^]^ We therefore urgently need to develop new approaches to overcome the problem of insufficient oxygen. BP DDSs with oxygen self‐supply strategies have been designed for this purpose. Pt, heme dimers, and manganese dioxide have been separately loaded onto BP as O_2_ suppliers to provide oxygen for PDT in real time, and all have exhibited enhanced photodynamic activity and antitumor capacity.^[^ ^51^, ^195^, ^196^ ^]^

#### 4.1.4 GT

GT is a treatment approach that introduces therapeutic genes into patients’ cells to correct abnormal, cancer‐causing genes or express specific proteins to prevent or treat related diseases.^[^ ^197^, ^198^, ^199^, ^200^, ^201^, ^202^ ^]^ To date, siRNA and the CRISPR/Cas9 system (clustered regularly interspaced short palindromic repeats, and CRISPR‐associated protein 9) have been widely used to control the regulation of gene expression. The development of GT depends on nanotechnology that involves the fabrication of powerful nanocarriers for the efficient intracellular delivery of therapeutic DNA/RNA molecules to prevent nuclease‐induced degradation and improve pharmacokinetics.^[^ ^203^, ^204^ ^]^

**Figure 14:**
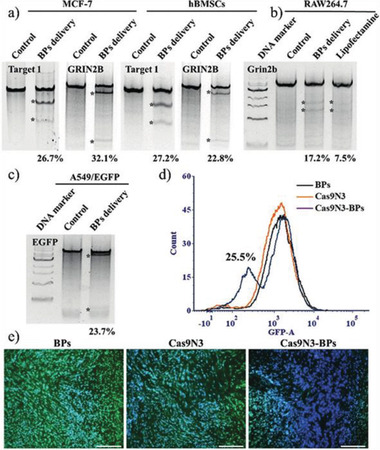
In vitro and in vivo genome editing and gene silencing of Cas9N3–BPs. a) Surveyor assays of MCF‐7 and hBMSCs cells treated with Cas9N3–BPs. b) Surveyor assays of RAW 264.7 cells treated with Cas9N3 via BPs delivery and Lipofectamine transfection. c) Surveyor assays of A549/EGFP cells treated with Cas9N3–BPs targeting EGFP. Controls: cells treated with only Cas9N3. d) Flow cytometry of A549/EGFP cells treated with BPs, Cas9N3, or Cas9N3–BPs. e) In vivo delivery of Cas9N3–BPs into A549/EGFP tumor‐bearing nude mice (scale bar: 100 mm). Reproduced with permission.^[^ ^56^ ^]^ Copyright 2018, Wiley‐VCH.

Designing and constructing an ideal DDS for GT is the key to expanding its practical application, to protect oligonucleotides from enzymatic degradation, promote cell uptake with high transfection efficiency, and intelligently release oligonucleotides from the DDS. Currently, plenty of efficient DDSs achieve efficient delivery of genetic materials, and 2D materials are also bringing their unique advantages to these nanoplatforms.^[^ ^200^, ^205^, ^206^, ^207^, ^208^, ^209^ ^]^ For example, Zhou et al. loaded CRISPR/Cas9 ribonucleoprotein onto a BPNs DDS for efficient GT involving genome editing and gene silencing.^[^ ^56^ ^]^ The cellular uptake of the Cas9N3–BPs DDS occurred via membrane penetration and endocytotic pathways, followed by endosomal escape and cytosolic releases of Cas9N3. The researchers also evaluated the effectiveness of the Cas9N3–BPs DDS in genome editing and gene silencing. As seen in **Figure** **14**a–d, in vitro studies showed higher editing and silencing efficiency than in parallel experiments at lower concentrations. In vivo therapeutic tests in A549/EGFP tumor‐bearing nude mice found significant reductions in the EGFP signals in frozen tumor sections around the injection site (Figure 14e). This model shows that the use of BP‐based DDS in GT should be further explored.

#### 4.1.5 SDT

SDT has emerged in recent years as a new cancer therapy. Similar to the mechanism of PDT, SDT excites sonosensitizer agents to generate ROS under ultrasound, and ultrasound cavitation often leads to increases in local temperature and pressure, thus promoting antitumor action.^[^ ^210^, ^211^ ^]^ In addition, SDT has unique advantages with respect to tissue permeability.^[^ ^212^, ^213^, ^214^ ^]^ But it is often limited by the instability, toxicity, biodegradability, and low ROS production yield of the sonosensitizer agents.^[^ ^214^, ^215^, ^216^ ^]^ In 2018, Ouyang et al. utilized BP's dual role as reductant and stabilizer to achieve the in situ growth of Au NPs on the surface of a BP platform.^[^ ^217^ ^]^ They found that ultrasound irradiation excited the electrons from the valence band (VB) to the conduction band (CB), and the electrons then transferred from BP to Au, and activated O_2_ to generate ^1^O_2_. This SDT reduced the survival rate of cells in vitro and caused apoptosis of cancer cells in vivo, suggesting for the first time the potential of BP as a sonosensitizer agent in SDT for BP‐based DDSs in tumor treatment. In 2020, Li et al. proposed SDT using pure BP as a model.^[^ ^215^ ^]^ However, this only proved BP's ability for SDT rather than as a DDS. It demonstrated that BP as a piezoelectric material produces •OH under ultrasound excitation, caused by piezoelectric polarization making the conduction band of BP more negative than that of O_2_/•O_2_^−^, while its valence band is more positive than that of H_2_O/•OH.

### 4.2 Bimodal Cancer Therapy

Above we discussed the success of BP‐based DDSs in monomodal cancer therapy. However, the shortcomings of monotherapy, such as instability, materials degradation, low efficiency in reaching action sites, and major side effects, have prompted a shift in focus toward combination therapy. Numerous clinical and exploratory studies have found the biggest impediment of a single treatment modality to be its ineffectiveness against subpopulations of cancer cells.^[^ ^218^, ^219^ ^]^ Because monomodal cancer therapy is incapable of eliminating the whole tumor, it cannot prevent metastasis, which arises primarily because heterogeneous tumor tissue can contain subpopulations of cancer cells that are resistant to monotherapy.^[^ ^220^, ^221^, ^222^ ^]^ Cancer progression is marked by numerous successive mutations in a line of cells, resulting in multiple complex pathways, and the use of a single drug may be insufficient to achieve tumor regression,^[^ ^130^ ^]^ so combination therapies can lead to better treatment outcomes. When BP‐based DDSs are used, their remarkable photothermal conversion capability and high extinction coefficient make PTT a brilliant candidate for synergistic cancer therapy, so it is included in almost all combination therapies. In what follows, we review in detail bimodal synergistic cancer therapies using BP‐based DDSs.

#### 4.2.1 PTT Combined with Chemotherapy

Although chemotherapy has achieved a certain degree of success in the treatment of cancer, monotherapy has deficits that limit its long‐term development and application, including poor drug specificity and targeting, low cellular uptake efficiency, and drug resistance caused by long‐term use. PTT is a mild physical hyperthermia strategy with some effectiveness but also ineluctable deficiencies. When these two therapies are combined, though, they can address each other's drawbacks. For example, PTT is able to completely eradicate primary tumors but fails to kill metastatic tumor cells, whereas chemotherapy is a systemic treatment paradigm for killing both.^[^ ^223^ ^]^ Bimodal synergistic cancer therapy that combines PTT with chemotherapy is now well documented as an effective strategy.

Among a number of reports on the application of BP‐based DDSs in PTT–chemotherapy bimodal synergistic therapy, the mechanisms that lead to enhanced therapeutic effectiveness can be divided into three categories. The main mechanism is based on PTT‐induced hyperthermia, which can enhance drug uptake by tumor cells. It has been demonstrated that mild hyperthermia (39–43 °C) can distort the junctional integrity of endothelial cells, which in turn increases the vascular permeability within the tumor. Alternatively, it increases the membrane fluidity and induces the denaturing of membrane proteins, thereby altering membrane permeability.^[^ ^224^, ^225^, ^226^, ^227^, ^228^, ^229^, ^230^, ^231^, ^232^, ^233^, ^234^, ^235^, ^236^, ^237^, ^238^, ^239^, ^240^ ^]^ Gao et al. found DOX‐loaded BP–DOX@PDA–PEOz to be more cytotoxic to tumor cells than DOX‐loaded BP–DOX@PDA–PEG, due to better cellular uptake of BP–DOX@PDA–PEOz.^[^ ^46^ ^]^ Using PTT with a BP–HSA–PTX DDS, Wang et al. assessed the cellular uptake behavior at 42.5 ± 0.5 °C by adjusting the power density of a NIR laser.^[^ ^45^ ^]^ BP–HSA–PTX exhibited higher fluorescence intensity with NIR irradiation than without, indicating that NIR light treatment enhanced the cellular uptake of their BP‐based DDS used with PTT. They also explored the efficiency of combination therapy in vitro and in vivo. A BP–HSA nanoplatform under NIR showed low cytotoxicity even at a high concentration of 0.5 mg mL^−1^. BP–HSA–PTX without NIR showed reduced cell viability due to the effect of PTX, but when NIR irradiation was added, the antitumor effect increased significantly. This enhancement was attributed to the synergistic effect of PTT and chemotherapy, caused by hyperthermia‐enhanced cellular uptake.

**Figure 15:**
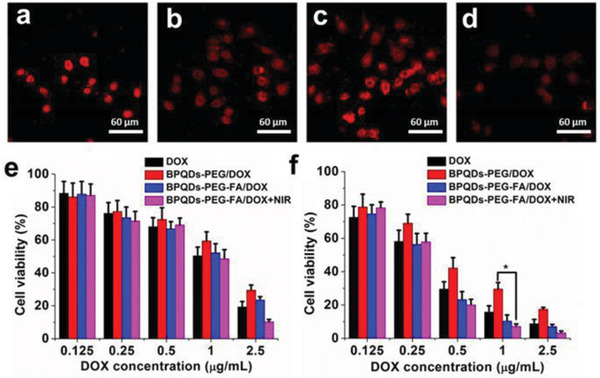
Confocal images of HeLa cells incubated with a) free DOX, b) BPQDs–PEG/DOX, c) BPQDs–PEG–FA/DOX, and d) BPQDs–PEG–FA/DOX+free folic acid after incubation for 2 h. Relative viability of HeLa cells after treatment with DOX and different NPs for e) 24 and f) 48 h. Reproduced with permission.^[^ ^81^ ^]^ Copyright 2019, Molecular Diversity Preservation International.

Another mechanism is to improve chemotherapy targeting, which leads to an increase in drug accumulation at the tumor site. Poor specificity and targeting often causes drugs to be rapidly cleared from the blood, causing side effects for normal cells and reducing the effectiveness of antitumor treatment.^[^ ^231^ ^]^ Employing a targeted DDS allows oncologists to use drugs in lower doses, reducing their cytotoxic effects while increasing efficacy. To promote therapeutic efficiency and construct specific targeted delivery systems, Luo et al. introduced FA with targeting capability into a BP platform, then studied the cellular uptake behavior of free DOX, BPQDs–PEG/DOX, and BPQDs–PEG–FA/DOX.^[^ ^81^ ^]^ Red fluorescence (indicating DOX) was observed for all groups after 0.5 h of incubation, demonstrating the successful internalization of these NPs. But after 2 h, the fluorescence intensity in cells with BPQDs–PEG–FA/DOX was significantly stronger than in others (**Figure** **15**a–d), suggesting that FA conjugation improved drug binding and uptake. An MTT assay indicated the highest therapeutic effect, showing that the active tumor‐targeting capacity of the FA ligand resulted in more accumulation of DOX (**Figure** **15**e,f).

Similarly, Tao et al.^[^ ^72^ ^]^ found a higher cellular uptake of DOX by BP–PEG–FA Nanosheets, and the in vitro cellular toxicity of BP–PEG–FA/DOX nanosheets was greater than that of BP–PEG/DOX nanosheets. Luo et al. demonstrated that a targeted FA carrier yielded better drug concentration at the tumor site, enhanced drug absorption, and improved treatment efficiency.^[^ ^81^ ^]^ Zhang et al. modified hyaluronic acid (HA) on BP‐based DDS (designated as BMH) to achieve active targeting capability.^[^ ^68^ ^]^ Although both BP and BMH can have a passive EPR target effect because they are nanosized, BMH added active targeting capability by modifying HA, causing greater accumulation of BMH in the tumor site than BP could achieve. PTT with BMH also effectively killed tumor cells and improved the release of MTX from BP; as a result, the BMH + NIR treatment group significantly inhibited tumor growth whereas groups using only BP + NIR or MTX–sol still experienced rapid growth in tumor volume.

Prolonged drug release time is another mechanism for enhancing PTT–chemotherapy bimodal therapy. The drugs combine with the BP carrier through interaction force and are transported to the tumor site. BP‐based DDSs can act as a “depot” for sustained, long‐term drug release. Liu et al. used a novel 2D SERS substrate of BP–AuNPs to deliver DOX.^[^ ^70^ ^]^ The in vitro chemo‐photothermal efficacy of DOX‐loaded BP–AuNPs was ≈90% when the DOX concentration was 6 µg mL^−1^. Along with a low tumor growth rate in vivo compared with single therapeutic treatment, the results suggested that chemo‐photothermal therapy based on this BP DDS exhibited excellent ability to inhibit tumor cells. A distribution study of DDS using grafted NIR fluorescent dye clarified the mechanism in this type of bimodal therapy, the signals sustainably increased during 48 h of observation, indicating a long retention time. A BP@hydrogel‐based DDS loaded with DOX also exhibited much better tumor ablation than free DOX by this mechanism.^[^ ^92^ ^]^ Dynamic changes in fluorescence were measured at 1, 12, and 24 h after irradiation. There was a strong free DOX signal at 1 h, but this disappeared within hours, whereas the group of BP@hydrogel samples exhibited localized drug distribution around the tumor site and more sustained release over 12 h. A study of BP–GEM–GEL found that the hydrogel led to the formation of a “depot” at the tumor site; the GEM (gemcitabine) was continuously released to the tumor and rapidly diffused into the surrounding tissues.^[^ ^93^ ^]^

#### 4.2.2 PTT Combined with PDT

PTT and PDT are the two main types of noninvasive phototherapy techniques for treating tumors. However, the hydrophobicity of photosensitizers, the highly hypoxic nature of the tumor microenvironment, the inevitable depth‐dependent decrease in photon intensity, and the nonuniform heat distribution within tumor tissues result in low ROS production efficiency and limited therapeutic effect. It is worth noting that the mild hyperthermia caused by PTT can promote the cellular uptake of photosensitizers and accelerate blood flow to increase the vascular oxygen saturation, which can enhance PDT efficacy.^[^ ^232^, ^233^ ^]^ Synergistic cancer therapy is a promising way to utilize these benefits. The two most common strategies for combining PTT and PDT are to use single‐laser irradiation to realize PTT and PDT, or to use two different lasers.

BP's completely unique nanostructure can receive PDT and PTT under dual wavelengths. Zhang et al. presented the first combined application of PDT and PTT using BPQDs.^[^ ^226^ ^]^ Their in vitro and in vivo assays exhibited excellent tumor‐inhibition efficacy by integrating PTT via a NIR laser at 808 nm and PDT at 660 nm. Most of the photosensitizer molecules used for PDT are excited by visible or UV light, which have low penetration depths in biological tissues. UCNPs have been used with excitation by a NIR laser emitting visible or UV light to generate ROS by PDT. For example, Dibaba et al. constructed a UCNP‐BPNS DDS then used it to achieve enhanced PDT under irradiation with a 980 nm laser;^[^ ^53^ ^]^ nearly 50% of the tumor cells were killed with NIR, but none dead without NIR. In addition to PDT at 980 nm, PTT at 808 nm also showed excellent anticancer effects. Using both wavelengths, the system worked as a form of bimodal synergistic PTT–PDT therapy.

**Figure 16:**
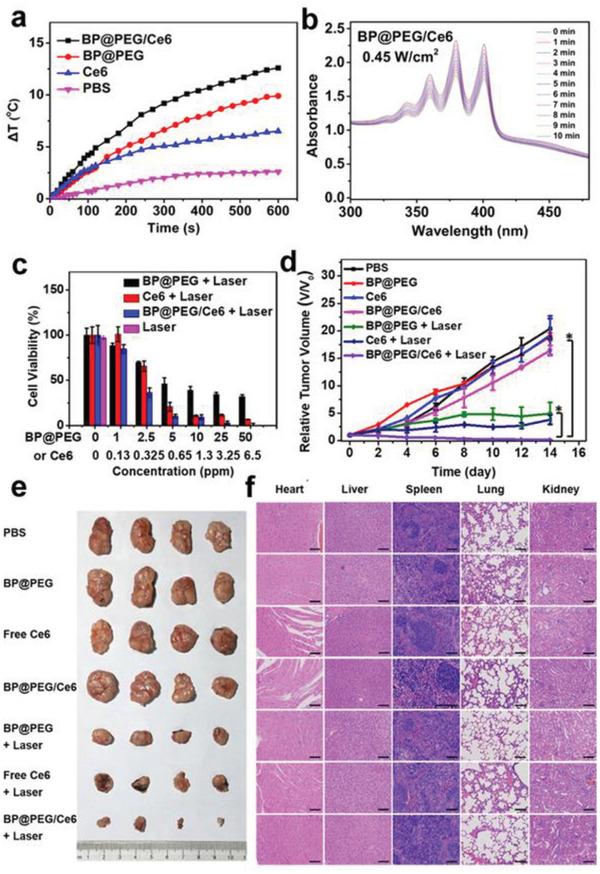
a) Photothermal heating curves of BP@PEG NSs (70 ppm), Ce6 (9 ppm), BP@PEG/Ce6 NSs (BP@PEG, 70 ppm), and PBS solutions under 660 nm laser irradiation (0.65 W cm^−2^, 10 min). b) Time‐dependent absorption spectra of BP@PEG/Ce6 NSs solution under 660 nm laser irradiation (0.45 W cm^−2^). c) Relative viabilities of HeLa cells after treated with BP@PEG NSs, Ce6, and BP@PEG/Ce6 NSs at different concentrations of BP@PEG NSs (1, 2.5, 5, 10, 25, and 50 ppm) or Ce6 (0.13, 0.325, 0.65, 1.3, 3.25, and 6.5 ppm) with irradiation (660 nm, 0.65 W cm^−2^, 10 min). d) Tumor growth curves of tumor‐bearing nude mice with various treatments (*n* = 4, mean ± SD, *P < 0.01). e) Photographs of tumors collected from the sacrificed mice. f) H&E stained histological images of major organs after 14 days treatment (scale bar, 100 µm). Reproduced with permission.^[^ ^44^ ^]^ Copyright 2018, American Chemical Society.

For patient comfort and operational convenience, a thoroughly studied strategy that has higher therapeutic efficacy is to combine these two phototherapy methods in a single beam. In 2017, Yang et al. assembled Fe_3_O_4_ and Au on BP (BPs@Au@Fe_3_O_4_).^[^ ^49^ ^]^ Due to the PDT of BP, the PTT of Au nanoparticles, and the tumor‐targeting and MRI‐guiding ability of Fe_3_O_4_, this nanoplatform exhibited remarkably enhanced therapeutic effects and greater selectivity than traditional remedies. The team applied a 650 nm beam to irradiate a BPs@Au@Fe_3_O_4_ composite using both PTT and PDT. In 2018, another of Yang et al. decorated Ce6 on a BP DDS to achieve synergistic cancer therapy with PDT via Ce6 and with PTT via BPNs under a single 660 nm laser.^[^ ^44^ ^]^ The as‐prepared BP@PEG/Ce6 nanosheets possessed the highest photothermal conversion efficiency (**Figure** **16**a) and achieved slow release of Ce6 for a prolonged PDT therapeutic time in vitro (Figure 16b). Analysis of the phototherapeutic effects in vivo found 64% of the cells had been killed, which was much higher than with BP@PEG Nanosheets (30%) or free Ce6 (34%) (Figure 16c). The material demonstrated great potential for PTT/PDT synergistic therapy by single laser irradiation in a clinical setting (Figure 16d–f). In 2019, this group prepared BP Nanosheets functionalized with both Ce6 and TPP, finding that the addition of TPP enabled the system to target mitochondria, which further enhanced its ability to eliminate a tumor.^[^ ^75^ ^]^

To resolve the problems of insufficient ROS generation and suboptimal therapeutic efficacy, we can use electromagnetic near‐field enhancement via plasmonic metals, based on a combination of oxygen‐independent therapeutic strategies like PTT. Zhang et al. adsorbed AuNPs on BP to increase the ROS production of BP nanosheets by maximizing the local field enhancement of AuNPs, at the same time enhancing the light absorption of the BP nanosheets to promote a photothermal effect by localized surface plasmon resonance.^[^ ^73^ ^]^ The results showed that the system had an enhanced inhibitory effect on tumor cells both in vitro and in vivo.

#### 4.2.3 PTT Combined with GT

As discussed above, GT uses nucleic acid polymers to prevent and treat various diseases, and this approach has attracted substantial attention. SiRNA plays a major role in GT by regulating the expression of specific genes to reduce the translation of corresponding mRNA.^[^ ^200^, ^234^ ^]^ However, research on GT and the applications of siRNA is still in the experimental and preclinical stages, and it is severely limited by the rapid enzymatic degradation of oligonucleotides under serum conditions and the low efficiency of intracellular uptake into cells.^[^ ^235^ ^]^ The design and construction of a highly efficient, nontoxic gene DDS is considered a promising solution, and 2D‐BP is a fine candidate, given its good biocompatibility and large surface area. Another strategy to improve the anticancer efficiency of GT is to combine it with PTT. By increasing the tumor cell uptake of genes and accelerating gene release from DDSs via hyperthermia, PTT can cooperatively enhance GT and lead to more efficient gene delivery.^[^ ^193^, ^236^ ^]^ GT can in turn enhance PTT by specifically inhibiting heat shock protein expression and reducing the resistance of cancer cells to heat damage.^[^ ^202^, ^237^ ^]^ These cooperative interactions can produce a remarkably synergistic therapeutic effect.

The first research on an siRNA‐loaded BPQDs DDS for gene therapy was conducted by Yin et al.^[^ ^54^ ^]^ Specific siRNAs that can target the LSD1 gene (which is closely related to the pluripotency and proliferation of cancer stem cells) were delivered into human ovarian teratocarcinoma PA‐1 cells via a BPQDs@PAH core–shell DDS for GT. PA‐1 cells treated with the BPQDs@PAH/siRNA exhibited the strongest Cy3 fluorescence signals, with the highest corresponding transfection efficiency (92.7%), indicating efficient delivery. They also found remarkable inhibition rates for relative mRNA and protein expression levels in the target LSD1 gene in vitro. Sufficient suppression was achieved in tumor cells treated with BPQDs@PAH/siRNA (62.1%), which was attributable to GT, but a more effective inhibition rate (over 80%) was observed with the combination of BP‐QDs@PAH/siRNA and 808 nm NIR light, indicating a synergism between the GT and PTT in the presence of BPQDs.

BPNs can also be loaded with siRNA on a DDS. The first report on BPNs used for GT was published in 2018.^[^ ^57^ ^]^ The research team showed that BP–PEI nanosheets could promote the cellular uptake of siRNA, and the resulting BP–PEI–siRNA complex successfully escaped from endosomes, as required for effective gene silencing. They further found BP–PEI–siRNA downregulated cancer‐relevant protein expression; ≈80% of the expression was suppressed with a 200 × 10^−9^ m siRNA dose, but there was no obvious inhibition of naked siRNA. The cancer cell growth also be controlled about 44%, in particular, 64% was inhibited under 808 nm irradiation. These results were also found in vivo, proving that the combination of PTT and GT yielded excellent tumor ablation.

#### 4.2.4 PTT Combined with CDT

CDT uses a Fenton‐like reaction to produce hydroxyl radicals by the catalysis of endogenous hydrogen peroxide to oxidize tumor cells, often requiring the introduction of a Fenton‐like reaction catalyst such as Cu^2+^, Ag^+^, or FePt. Without additional conditions such as light and ultrasound, only H_2_O_2_ can be used to eliminate tumors all the time, so it is often combined with PTT and other therapies for collaborative antitumor action. In 2020, Hu et al. incorporated Cu^2+^ onto BPNs to combine PTT and CDT.^[^ ^60^ ^]^ The Cu^2+^ enhanced the PTT of the BP@Cu nanostructures while also enabling the CDT, an approach rarely used in BP‐based cancer therapy. The Cu^2+^ promoted the degradation of BP via a redox reaction to achieve rapid degradation. At the same time, the Cu^2+^ was reduced to Cu^+^, which reacted with local H_2_O_2_ via a Fenton‐like reaction to generate •OH for CDT. Their report provides new ideas for cancer treatment with BP‐based DDSs and broadens its application in tumors. Subsequently, Su et al. assembled a nanosystem that included BPNs coated with PDA and Ag NPs, placing this in a decellularized small intestinal submucosa extracellular matrix (dSIS–ECM) for postoperative skin cancer therapy.^[^ ^238^ ^]^ The BP‐coated PDA played a part in PTT and further accelerated the Ag in catalyzing the conversion of H_2_O_2_ into •OH. The as‐obtained dSIS–BPNs–PDA@Ag not only killed tumor cells but also inhibited the recurrence of residual cancer cells. In the absence of NIR, CDT with Ag reduced the recurrence rate from 100% to 67%, and to only 33% after additional NIR.

### 4.3 Multimodal Synergistic Cancer Therapy

Although the above‐mentioned bimodal synergistic therapy can greatly improve the efficiency of antitumor treatments, the cooperative interactions that characterize trimodal synergistic cancer therapy can achieve even more remarkable effects through the simultaneous targeting actions of three therapeutic agents. Triple therapy achieves concurrent inhibition of multiple pathways of tumor cells, not only improving treatment efficiency but also minimizing the development of drug resistance. Further, this trimodal therapy achieves optimal anticancer efficacy with lower drug doses, thereby minimizing side effects. In this section, we review four representative types of trimodal synergistic therapies with these integrated advantages.

#### 4.3.1 PTT/PDT/Chemotherapy

**Figure 17:**
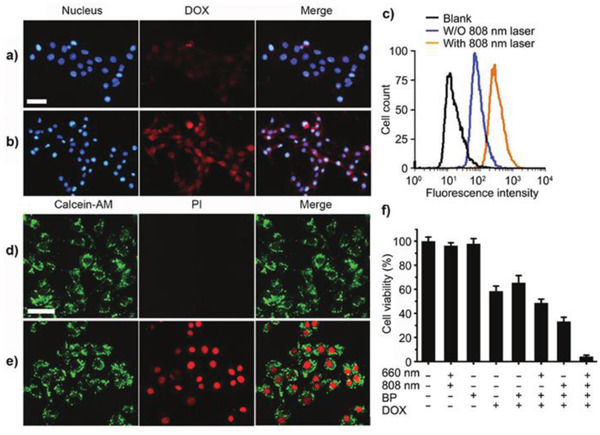
a) Fluorescence images of 4T1 cells incubated with BP–DOX without or b) with 808 nm laser irradiation (0.8 W cm^−2^). c) The intracellular fluorescence was analyzed through flow cytometer. d) Fluorescence images of 4T1 cells treated with BP–PI without or e) with 808 nm irradiation (0.8 W cm^−2^). f) MTT assay of 4T1 cells under different treatments (scale bar = 50 µm). Reproduced with permission.^[^ ^41^ ^]^ Copyright 2016, Wiley‐VCH.

When PDT, PTT, and chemotherapy are combined, the resulting synergistic effects can boost their antitumor efficacy. One of the main reasons is that the local, continuous hyperthermia caused by PTT promotes the release of drugs from DDSs to enhance the chemotherapy, another mechanism is the PTT also increased membrane permeability to promote the PDT. DOX targets the nucleus, whereas PDT usually causes oxidative damage to the organelles, so cell membrane permeability enhanced by PTT provides more opportunities for chemotherapy and PDT to get inside cancer cells.^[^ ^239^, ^240^ ^]^ As described in Chen et al.’s study about intracellular drug release behavior (**Figure** **17**a,b), a weak fluorescent signal occurred from the endocytosis of BP–DOX nanocomposites, but after the addition of an 808 nm NIR laser, a significant increase in red fluorescence occurred.^[^ ^41^ ^]^ Flow cytometer results confirmed that the intracellular fluorescence intensity of DOX after irradiation with the 808 nm laser increased fourfold compared with BP–DOX in the dark (Figure 17c), demonstrating the enhanced efficacy of chemotherapy with PTT. Next, they adopted two dyes to investigate changes in membrane permeability. It is well known that propidium iodide (PI) enters a cell and binds to the nucleus after the cell membrane has broken down or become less permeable, then emits red fluorescence, while calcein‐AM can enter the cytoplasm of living cells and emit green fluorescence. As Figure 17d,e, their study showed that cytoplasm was stained by calcein‐AM blue little red fluorescence from PI was observed in the nuclear area, when cells were irradiated under a 808 nm laser for 5 min, both performed strong fluorescence, suggesting that the PTT efficiently increased cell membrane permeability. Finally, through the combined action of several mechanisms, BP–DOX under simultaneous irradiation at 660 and 808 nm showed the strongest tumor cell inhibition capacity (95.5%) (Figure 17f).

Wu et al. also developed a BP‐based DDS synergistic therapy using PTT/PDT/chemotherapy to effectively inhibit chemotherapy‐resistant tumor cells.^[^ ^67^ ^]^ The p53 gene is believed to be closely related to cell drug resistance, as mutations in the p53 gene are often associated with drug‐resistant tumors, so reducing the mutation level of the p53 gene is an effective way to solve drug resistance.^[^ ^241^, ^242^, ^243^, ^244^ ^]^ In one study, DOX was loaded on a BP DDS, along with phenethyl isothiocyanate (PEITC), which can decrease mutant p53 levels and enhance chemotherapy drug sensitivity. The results showed that the expression of mutant p53 was significantly inhibited in the presence of PEITC, and DOX further reduced the survival rate of cancer cells. BPNs–PDA–PEG–PEITC/DOX +660 nm +808 nm showed the best treatment effect, arising from the trimodal synergistic effects of PTT, PDT, and chemotherapy.

#### 4.3.2 PTT/PDT/GT

Although GT has made an indispensable contribution to the elimination of tumors, its poor specificity and low efficiency often lead to failure in application. An ideal carrier and a combination of PDT/PTT can address this deficiency. As a 2D material with excellent properties, BP not only can be used as a high‐quality DDS for GT but also is a very efficient photosensitizer for PDT and possesses NIR photothermal properties for PTT. Representative studies have shed new light on the design of multifunctional nanomaterials for trimodal synergistic therapy. For example, the polymer‐modified BPNs designed and synthesized by Chen et al. successfully delivered hTERT siRNA, which is important in cancer GT, and both PDT and PTT under different wavelengths of laser irradiation gave the system stronger antitumor efficacy.^[^ ^55^ ^]^ hTERT siRNA exhibited strong cellular uptake via this BP‐based DDS, and the degradation of the BP platform further promoted the release of hTERT siRNA, which ultimately improved the therapeutic efficiency of GT.

#### 4.3.3 PTT/Chemotherapy/GT

By the co‐encapsulation of a drug and siRNA into a photothermal nanocarrier, the combination of chemotherapy, GT, and PTT can be realized within a single nanostructure. In addition, the DDS can enhance the tumor cell uptake of the drug and siRNA, resulting in a remarkable trimodal synergistic therapeutic effect. For this reason, Zeng et al. loaded DOX and P‐gp siRNA on BPNs with aptamers (Apts), which bind specific target molecules.^[^ ^74^ ^]^ Combining the PTT of BPNs, chemotherapy with DOX, and GT with P‐gp siRNA, as well as the tumor targeting ability of Apts, this nanoplatform exhibited remarkable multimodal therapeutic effects by restricting cell proliferation in solid tumors in vitro and in vivo. This was attributable to the three effects of the DDS on DOX and siRNA: promotion of endocytosis, protection of siRNA, and effective co‐delivery of DOX and RNA.

#### 4.3.4 PTT/PDT/CDT

Integrated multicomponent and synergetic multi‐modal therapy would allow for an effective cancer therapy at the same time. Therefore, reasonable design and introduction of composite components to achieve mutual promotion is particularly important. Zhang and co‐workers developed BPQDs for PDT and FeOCl for CDT, and further ingeniously established Prussian blue (PB) and Mn^2+^ to catalyze H_2_O_2_ to release O_2_ within the solid tumor to achieve high PDT or CDT, and simultaneously introduced PAD combined PB for PTT.^[^ ^115^ ^]^ They proved CDT, PDT, and PTT that combined with multimodal imaging achieved a more significant synergistic therapeutic result compared to any single treatment modality alone.

#### 4.3.5 PTT/PDT/Chemotherapy/Immunotherapy

Immunotherapy activates the individual's own immune system to rid the body of foreign invaders such as cancer cells.^[^ ^245^ ^]^ One of the main benefits of immunotherapy is that it can distinguish between cancer cells and normal cells, then attack the diseased cells, reducing the damage to normal cells.^[^ ^246^ ^]^ However, immunotherapy can cause some side effects, such as high blood pressure, nausea, diarrhea, and fatigue, while PTT, PDT, and chemotherapy can also trigger the immune system. Immunotherapy combined with other therapies can effectively improve treatment efficiency and minimize side effects.^[^ ^247^, ^248^ ^]^ Cytotoxic T‐lymphocyte (CTL)‐associated protein 4 and programmed death 1 (P)/programmed death ligand 1 (PL) blockades are used as immunity‐stimulating agents to modulate the immunosuppressive responses within tumors and thereby improve cancer treatments. In 2018, Ou et al. loaded a drug (doxorubicin), a targeting agent (chitosan−polyethylene glycol), and cancer growth inhibitors (PL and siRNA) on BPNs to achieve potent chemo‐photoimmunotherapy of colorectal cancer.^[^ ^58^ ^]^ These multiple therapeutic effects were feasible in both C57BL/6 and Balb/c nude mouse models, and the survival period of the treated group was significantly prolonged.

#### 4.3.6 PTT/PDT/CDT/Immunotherapy

According to Yao et al.’s report, PTT is capable of inhibiting metastatic tumor growth by stimulating the host immune system to release tumor antigens into the tumor microenvironment and promoting the expression of tumor‐derived antigens to the T cells.^[^ ^249^ ^]^ For this reason, PTT is always combined with a cytotoxic T lymphocyte‐associated protein 4 (CTLA‐4) blockade to achieve better therapeutic results. Yao et al. constructed FePt/BP–PEI–FA nanoplatforms to achieve synergistic PTT/PDT/CDT and PTT‐enhanced immunotherapy. BP plays the role of PTT and PDT, and FePt catalyzes H_2_O_2_ to produce hydroxyl radicals, which have a high photothermal conversion rate and ROS yield. The result is not only a strong ability to kill cancer cells, but also a certain inhibitory effect on primary and metastatic tumors.

### 4.4 Other Cancer Therapies

In light of the enhanced treatment efficacy of trimodal synergistic therapies over bimodal approaches, attention has shifted to other combinations of interrelated therapeutic modalities based on their cooperative interactions. Gui's group proposed two DDSs with traditional Chinese medicine as an important component, which had a certain inhibitory effect on tumor growth through different mechanisms.^[^ ^48^, ^82^ ^]^

**Figure 18:**
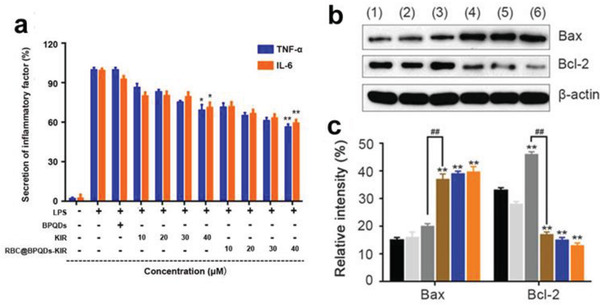
a) The TNF‐*α* and IL‐6 secretion of macrophages treated by PBS (negative control) and lipopolysaccharides (LPS) (positive control) was set as 0% and 100%, respectively. Data are mean ± SD (*n* = 3). ^*^ *p* < 0.05 versus LPS. ^**^ *p* < 0.01 versus LPS. b) The western blotting and c) semiquantitative analysis of Bax and Bcl‐2 expression in Hela cells treated with different scheme as above for 24 h. Data are mean ± SD (*n* = 3). ^**^ *p* < 0.01 versus control. ^##^ *p* < 0.01. Reproduced with permission.^[^ ^48^ ^]^ Copyright 2019, Informa UK Limited.

In 2019, Huang et al. inhibited tumor cells by combining chemotherapy with antiinflammatory therapy.^[^ ^48^ ^]^ It is well known that the tumor microenvironment, which consists of immune cells and inflammatory factors, has an important influence on tumor growth and resistance. In this microenvironment, the infiltration of immune cells promotes tumorigenesis, invasion, and metastasis. On the other hand, inflammation can attenuate the host's immune response against tumors. When foreign agents such as drugs and DDSs enter the tumor, they activate the immune system and cause inflammation. In addition, dying cells exposed to chemotherapy drugs also stimulate immune cells to produce inflammatory effects, leading to the development of drug resistance. Hence, antiinflammation is particularly necessary for removing tumors and preventing drug resistance. In Huang et al.’s research, RBC membrane camouflage was utilized to evade the immune system, and antiinflammation therapy was simultaneously adopted for specific and highly efficient antitumor treatment. Studies of the in vitro antiinflammatory effects showed that KIR, a bioactive antiinflammatory ingredient, and RBC@BPQDs–KIR substantially decreased the proinflammatory cytokines of TNF‐*α* and IL‐6 by 30.7% and 28.6%, respectively (**Figure** **18**a). And the in vitro antitumor performance found 33.4% cell viability was slightly decreased by RBC@BPQDs‐DOX/KIR. Research into the mechanism demonstrated that the combination of KIR could significantly inhibit Bcl‐2 expression and improve the proapoptosis protein Bax (Figure 18b,c), which synergistically enhanced the chemotherapeutic effects of DOX, and RBCm enhanced the permeability and retention effects of RBC@BPQDs‐DOX/KIR. The in vivo results were consistent with these findings, showing that DOX and KIR acted synergistically in RBC@BPQDs‐DOX/KIR, reducing the tumor cells’ resistance to death. This study provided a new way of thinking about tumor treatment and brought inflammatory therapy into researchers’ field of vision.

As mentioned earlier in this review, Shang et al. designed a platform of platelet‐membrane‐camouflaged BPQDs loaded with HED (PLT@BPQDs‐HED) and explored its antitumor effect.^[^ ^82^ ^]^ They also proposed cell autophagy and apoptosis as new antitumor mechanisms. Autophagy can protect organelles from damage while at the same time killing the tumor, and apoptosis is one mechanism of cell death. These two processes affect each other both positively and negatively. Shang et al. demonstrated the inhibitory effect of PLT@BPQDs‐HED on cancer cells by observing changes in cell survival rate in vitro and measuring the volume of cancer cells in vivo. TEM revealed a greater number of vacuoles (autophagosomes) in the PLT@BPQDs‐HED‐treated cells, indicating a stronger ability to induce autophagy. Measurement of the cell apoptosis rate demonstrated that more cell apoptosis had been induced. To determine the mechanism, they then measured relevant indicators such as mitochondrial membrane potential, intracellular ROS, and terminal deoxyribonucleotide transferase‐mediated dUTP nick‐end‐labeling (TUNEL)‐positive cells, demonstrating that the platform significantly enhanced tumor targeting and promoted mitochondria‐mediated cell apoptosis and autophagy in tumor cells.

### 4.5 Imaging‐Guided Cancer Therapy

Cancer imaging has generated a new need for the integrated diagnosis and treatment of antitumor DDSs. Examples of imaging‐guided cancer therapy using BP‐based DDSs are numerous due to the flexible loading of the functional molecule. BP itself has been reported to be capable of fluorescence as well as photoacoustic and photothermal imaging, and BP‐based DDSs have even more extensive imaging applications when they carry drugs.^[^ ^32^, ^77^, ^159^, ^182^, ^250^, ^251^, ^252^ ^]^

BP's large specific surface area results in DDSs with a wide range of imaging modes, among which fluorescence imaging is the most available through the simple loading of fluorescent dyes. NB dye can be immobilized onto BP via diazonium chemistry, forming NB@BPs with environmental stability and strong NIR fluorescence.^[^ ^65^ ^]^ Fluorescence images of MCF7 breast tumor‐bearing nude mice treated with NB@BPs exhibited considerable fluorescence signals up to 24 h after injection. The organic fluorescent dye rhodamine B (RdB) can also be loaded, allowing the reliable imaging of cancer cells.^[^ ^80^ ^]^ Confocal fluorescence images of Hep G2 and 4T1 cells in vitro have shown the possibility of fluorescence imaging in the cytoplasm. Ce6 has also been shown to be effective in fluorescence imaging, demonstrating strong fluorescence in tumor tissue.^[^ ^44^ ^]^ Tao et al. and Deng et al. loaded cyanine 7 and FITC onto BP nanosheets for in vivo NIR imaging and achieved desirable fluorescence imaging at a tumor site.^[^ ^72^, ^253^ ^]^

Due to its high extinction coefficient and excellent photothermal conversion efficiency, BP can be used for photothermal imaging. Both a BP nanosheet as the substrate and a hydrogel containing BP can distinctly increase the system temperature to make it suitable for photothermal imaging, and sometimes it can also be coated with PDA to improve the image resolution.^[^ ^42^, ^70^, ^74^, ^92^ ^]^ Deng et al. suggested that considering the above remarkable photothermal conversion performance and photothermal stability, BP could be a promising photothermal transducer for PA imaging.^[^ ^253^ ^]^ In vitro PA imaging yielded a strongly enhanced photoacoustic signal, and the maximum signal in vivo was 3.1 times higher than that of the precontrast image, which provided substantial evidence for the researchers’ assumptions.

Most recently, Hu et al. demonstrated a positron emission tomography (PET)‐guided combination cancer therapy by employing ^64^Cu^2+^ to label BP.^[^ ^60^ ^]^ PET, with its excellent quantitative capability, unlimited signal penetration, and high detection sensitivity, can be combined to detect the pharmacokinetics of drugs in vivo in real time. When PET imaging of saline and BP@Cu_0.4_@–PEG–RGD was compared, significantly smaller tumor size and volume was observed.

## 5 Biocompatibility of BP‐Based DDSs

Biocompatibility is an absolutely key factor for in vivo anticancer carriers, affecting their application and translation into clinical settings. Biocompatibility testing can be done in vitro or in vivo, with the former analyzing MTT, hemolysis, and antiphagocytosis, and the latter assessing blood biochemistry, pathological changes in tissues or organs, and immunotoxicity. As phosphorus is an essential element for living organisms, and BP easily degrades into harmless phosphates, it is highly suitable for use in biomedicine. In the construction of BP‐based DDSs, the BP is often loaded with materials that further improve biocompatibility, such as PEG or PDA. In what follows, we briefly review common methods for measuring platform biocompatibility.

### 5.1 Cytotoxicity

The 3‐(4,5‐dimethyl‐2‐thiazolyl)‐2,5‐di‐phenyl‐2‐H‐tetrazolium bromide (MTT) assay is the most common method of assessing biocompatibility. After co‐culturing a sample with cells for a period of time, the difference in MTT absorbance is measured to evaluate the cells’ biological activity. In most of the reports on BP‐based DDSs, MTT detection has shown their excellent biocompatibility. Chen et al. exposed 4T1, HeLa, L929, and A549 cells to different concentrations of BPNs, finding that the viability rate of the four types of cells at the same concentration was basically the same and remained ≈100%, indicating the low toxicity of BP even at a concentration of 200 µg mL^−1^.^[^ ^41^ ^]^ Wang et al. used U87MG cells to evaluate the cellular cytotoxicity of BP–HSA platforms and found that cell viability remained above 80% at a high concentration of 500 µg mL^−1^.^[^ ^45^ ^]^ Dibaba et al. demonstrated the good biocompatibility of a high concentration (800 µg mL^−1^) of UCNP–BPNS, with HeLa cells incubated after 24 h retaining almost 100% viability.^[^ ^53^ ^]^

Yang et al. explored an MTT assay of BPs@Au@Fe_3_O_4_ nanoparticles at a dose of 1 mg mL^−1^. After 24 h, the viability percentage had dropped but was still above 75%, and hardly any morphological changes were observed in the cells, indicating its good biocompatibility.^[^ ^49^ ^]^ Many other MTT analyses of BP substrates and DDSs have demonstrated the platforms’ low toxicity and good potential for biomedical applications. By studying the biocompatibility of BPHs, Xing et al. proposed and demonstrated a special role for hydrogels in improving the biocompatibility of BP‐based DDSs.^[^ ^42^ ^]^ They showed that the BP and the embedded structure of the drug in the hydrogel blocked direct contact with outside cells before reaching the designated location, thus inhibiting its potential toxicity.

### 5.2 Hemocompatibility

Hemocompatibility is another important indicator of biocompatibility. Most bioapplications of nanocarriers are administered intravenously, and hemolysis leads to hemoglobin release, which causes thrombosis, so hemocompatibility tests must be conducted. Fragmentation or aggregation of red blood cells indicates incompatibility. Typically, PBS and water are used as negative and positive controls, and the hemolytic rate of a sample is calculated from the absorbance detected by a UV–vis spectrophotometer at a wavelength of 540 nm. The standard value is 5%.^[^ ^117^, ^254^ ^]^ Yang et al., Liu et al., Ouyang et al., Huang et al., and others have confirmed the low (<5%) hemolytic rate of some BP‐based DDSs.^[^ ^44^, ^48^, ^49^, ^51^, ^70^ ^]^ Lv et al. found no visual evidence of red in PBS solutions mixed with UCNPs−BPS, and the highest hemolytic rate with 1 mg mL^−1^ was only 0.91%.^[^ ^52^ ^]^ Shang et al. found that even at a concentration of 2.0 mg mL^−1^, BPQDs or PLT@BPQDs elicited less than 1% hemolysis.^[^ ^82^ ^]^

**Figure 19:**
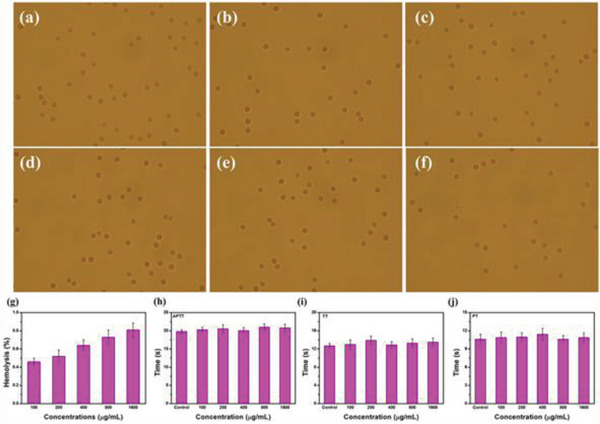
Morphology images of RBCs incubated with BPNs–PDA–PEG–PEITC at concentrations of a–f) 0 (negative control), 100, 200, 400, 800, and 1600 µg mL^−1^. g–j) Hemolysis, activated partial thromboplastin time (APTT), prothrombin time (PT), thrombin time (TT) assays of BPNs–PDA–PEG–PEITC with different concentrations. Reproduced with permission.^[^ ^67^ ^]^ Copyright 2019, Elsevier Ltd.

In their assessment of hemolysis caused by BPNs–PDA–PEG–PEITC or the DDS BPNs–PDA–PEG–PEITC/DOX, Wu et al. observed morphological changes in blood cells, hemolytic rate, and clotting time (**Figure** **19**).^[^ ^67^ ^]^ First, they found that after co‐culture with the samples, the blood cells in the platform retained their circular morphology without fragmentation or aggregation, but a few morphological changes were found in the DDS due to hemolysis by free DOX. Second, they determined that the hemolytic rate at a BPNs–PDA–PEG–PEITC concentration of 1600 µg mL^−1^ was only 0.81%, but it was higher with BPNs–PDA–PEG–PEITC/DOX. Finally, the clotting times for both platforms and the DDS were close to the control group's time, indicating excellent hemocompatibility and showing that DOX had no effect on clotting time.

### 5.3 Antiphagocytosis Effect

Biocompatibility can also be measured by checking whether a DDS stimulates immune responses that induce engulfment by macrophages once it enters the body. Shang's team and Huang's team assessed the antiphagocytosis effect of BPQDs, PLT@BPQDs, and RBC@BPQDs.^[^ ^48^, ^82^ ^]^ The samples were modified with RhB (red fluorescence), then their antiphagocytosis ability was evaluated by detecting the fluorescence intensity of RAW264.7 macrophages. Both studies found that the macrophages treated with BPQDs–RhB emitted intense red fluorescence signals, indicating a strong phagocytic effect, whereas the fluorescence intensity of the BPQDs groups after PLT and RBC membrane coating was weak, demonstrating that the vesicles enabled the BPQDs to evade the immune system, reducing their clearance from the body.

### 5.4 In Vivo Toxicity

The toxicity of materials for tissues and organs after they enter the body needs careful evaluation. Chen et al. and Ouyang et al. studied in vivo toxicity by injecting BP, then measuring blood biochemistry (RBCs, white blood cells, platelets, hemoglobin, and blood cell densities) as well as liver and kidney function markers (alanine aminotransferase, aspartate amino‐transferase, blood urea nitrogen, and creatinine).^[^ ^41^, ^51^ ^]^ The results showed that the blood parameters were all within normal ranges, and the index was not different from that of the healthy group, indicating the good biocompatibility of BP in vivo.

**Figure 20:**
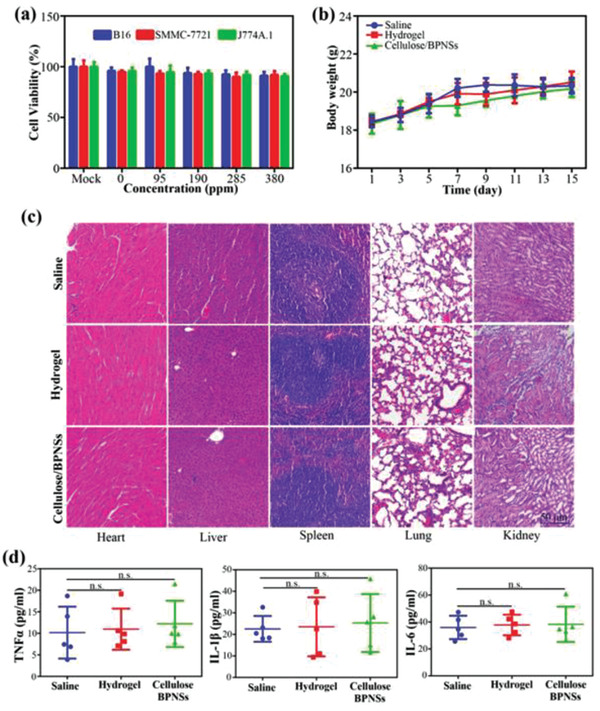
Toxicity assays. a) In vitro toxicity assays. Relative cell viability of B16, SMMC‐7721, and J774A.1 cells after incubation with Mock (no treatment) and hydrogels without BPNSs or with BPNS concentrations of 95, 190, 285, and 380 ppm for 24 h. b) Body weight was monitored on days 1, 3, 5, 7, 9, 11, 13, and 15. c) Hematoxylin and eosin (H&E) staining of the heart, liver, spleen, lung, and kidney on day 15. d) Immunotoxicity assays in vivo for protein levels of TNF‐*α*, IL‐1*β*, and IL‐6 in the serum of C57BL/6 mice after indicated treatments. Data were shown as mean ± SD, Student's *t*‐test, *n* = 5, n.s. means *p* > 0.05, nonsignificant. Reproduced with permission.^[^ ^42^ ^]^ Copyright 2018, Wiley‐VCH.

Zeng et al. and Shao et al. imaged major organs (heart, liver, spleen, lung, and kidney) to assess the in vivo toxicity of BP‐based DDS, finding no obvious pathological changes in any of the organs.^[^ ^43^, ^74^ ^]^ Xing et al. subcutaneously injected ground‐up cellulose‐based hydrogels with and without BP into mice to determine their toxicity in vivo (**Figure** **20**).^[^ ^42^ ^]^ First, they monitored weight changes in the mice for two weeks and found that the presence or absence of BP had no effect on their growth. Then they looked for histological changes in the main organs and found no obvious injuries. They also evaluated the immunotoxicity of the cellulose/BPNS hydrogel by measuring the protein expression levels of inflammatory cytokines in serum and found no significant changes, indicating it did not induce inflammation in vivo. All of these results suggested that this BP‐based DDS had high biocompatibility and biosafety.

## 6 Conclusions and Outlook

The research and development of anticancer drugs, cancer therapies, and cures continue to fuel massive, intense, and prolonged efforts throughout the globe. As a 2D nanomaterial, BP, with its excellent PDT/PTT capability, large specific surface area, fold structure, good biodegradability, and high biocompatibility, has been the subject of numerous studies in recent years to counteract cancer or deliver anticancer drugs in DDSs.^[^ ^33^, ^34^, ^35^, ^36^ ^]^ The outstanding PDT/PTT capability of BP‐based DDSs has increased their antitumor capacity without the additional loading of photosensitizer and photothermal agents. In additional, their large specific surface area has expanded the possibilities for drug loading, enabling the combination of more drugs. Although BP‐based DDSs are small, their fold structure ensures a high surface area that enhances their delivery capabilities. Along with their good biological compatibility and biodegradability, they are perfectly suited for use in biomedical fields. This review has surveyed BP‐based DDSs for cancer therapies, providing a detailed summary of research into common construction strategies, drug release modes, tumor therapy applications, and assessment of biocompatibility.

In the construction of an optimal DDS that is stable and efficient, the choices of platform and drug load determine the DDS's application. Many research groups covered in this review have developed effective methodologies for synthesizing BP‐based DDSs. Most of the early procedures focused on bare BPN platforms, using BP's negative surface charge to combine with positively charged chemotherapy drugs such as DOX and MTX through electrostatic interaction. Subsequently, a few studies of BPQD‐based platforms appeared.

As synthesis techniques and skills have improved, more has been required of DDSs.^[^ ^95^ ^]^ Components for drug loading and delivery to tumor sites have become diverse; in addition to traditional chemotherapy drugs, gene components, nanoparticles, and photosensitizers have been incorporated into the diagnosis and treatment of cancer. As a bare BP substrate is unable to cope with the rich variety of drugs, modified BPNs have emerged. Presently, polymerization and grafting/coating approaches are the most common techniques for giving BPNs platforms the capacity to change electrical properties, increase functional groups/active sites, and improve stability, making BP‐based DDSs more abundant and multifunctional.

As the unique advantages and extensive applications of hydrogels in biomedicine have emerged in recent years, these also have been introduced into the construction of BP‐based DDSs.^[^ ^83^ ^]^ The coating and viscosity of hydrogels have greatly improved the stability and biocompatibility of DDSs and extended the range of their application. Although numerous diverse DDSs have been created using various synthesis approaches, the following findings have emerged: 1) among the various BP platforms, BPNs is the most popular due to its physicochemical advantages; 2) BPNs modified with PEG constitute the majority of modified materials; 3) electrostatic interaction is the main strategy for drug loading.

Although these developments are encouraging and show great potential, major challenges remain to be solved before these DDSs can move from experimentation to clinical use. 1) The size and thickness of BPNs are still not completely controllable during the exfoliation process.^[^ ^255^ ^]^ In addition, the cost of preparing BPNs is too high and the yield too low, and it is difficult to prepare them in high concentration and with multiple layers.^[^ ^256^ ^]^ The insufficient reproducibility of BP substrates, along with deviations in the constructed DDSs, presently prevent the move to clinical applications.^[^ ^9^ ^]^ While continuing to enrich the components of BP‐based DDSs, such as coatings on BP platforms, drug types, and various functional molecules, researchers need to focus on the preparation of 2D‐BPNs to precisely control their sizes, thicknesses, and surface coatings so that batch‐to‐batch variation is minimized. Large‐scale production is another challenge waiting to be solved. It is well known that the size and thickness of 2D materials are related to a given sample's properties, so it is difficult to achieve a balance between manufacturing scale‐up and product quality.^[^ ^9^ ^]^ To solve this problem, potential future research could begin by addressing two angles. First, more preparation methods need to be explored. Most exfoliation is still based on solvents, which usually lead to a wide variety of products. Microwave‐ and electricity‐mediated methods could be explored, as they are controllable and adjustable. Second, standardized manufacturing protocols should be developed. 2) Electrostatic interactions are still the most common method of building DDSs. Although convenient, this approach inevitably brings stability problems. Changes in the surrounding environment, pH, temperature, charges, etc., will to some degree disrupt the bonds between the DDS and the drug.^[^ ^257^ ^]^ Therefore, methods that combine strong bonding and ingenious design are needed, such as bonds that can be adjusted or reversed by external conditions. 3) Presently, most DDSs are limited to delivering anticancer drugs or materials, and few are capable of multifunctional applications. As the requirements of people on DDSs increase, single functionality can no longer meet patients’ actual needs, so other auxiliary or independently functional materials must be designed. These include dyes, contrast agents, and materials containing elements with a high atomic number, such as W, Au, Bi, and Pd, which have outstanding X‐ray absorption coefficients that make them suitable for use as CT imaging contrast agents to provide high‐resolution 3D structural details of the whole body; these would enable simultaneous imaging, detection, and diagnosis.^[^ ^9^ ^]^

An excellent DDS should have zero premature release and accurate stimulus response. Stimuli‐responsive BP‐based DDSs respond to pH, NIR or GSH. The differences between the pH of tumor microenvironments and the normal physiological environment support the feasibility and convenience of this kind of stimulus response, so it has drawn the most research.^[^ ^102^ ^]^ In addition to the mechanisms that pH can bring about in all DDSs (protonation of drugs, decomposition of coatings or capsules, and destruction of bonds), its unique way of controlling drug release from BP‐based DDSs is to induce the degradation of BP, leading to decomposition of the substrate and promotion of drug release. Drug release via NIR stimulation can be divided into two types, PTT triggered and PDT triggered, with the majority falling into the first category. Given the characteristics of BP, even without additional photothermal materials the DDS itself can trigger a photothermal reaction, converting NIR into heat that promotes drug release, and thereby allowing simultaneous drug release and PTT. PDT of BP can also be utilized to cause degradation of a BP platform by ROS.

The stability of DDSs is essential for translating promising nanoplatforms into treatments for patients in clinical settings. To date, BP‐based DDSs have basically only few response modes, but since there are numerous scenarios requiring the regular release of drugs, more response modes, such as ROS, ATP, enzymes, microwave, are needed to enrich the scope of DDS applications. One approach is to take advantage of physiological cues that are typical of cancer cells and tumors, using a biological stimuli‐response mechanism such as reducing conditions, low pH, and biomolecules to trigger drug release. Notably, a significant problem is that individual differences between patients, such as the temperature and pH of tumor sites, affect drug release efficiency, so it is important to be able to regulate and optimize parameters by applying external response. For example, physical stimuli‐responsive such as thermo‐, magnetic‐, electrical‐, chemical stimuli‐responsive such as redox.^[^ ^97^ ^]^ Since individual stimuli‐responsive methods have been reasonably well validated, experiments to find dual‐responsive, tri‐responsive, or even tetra‐responsive approaches should be explored with BP‐based DDSs.

BP‐based DDSs offer tremendous opportunities for the design of anticancer treatment strategies involving mono‐, bi‐, and multimodal therapies. Although monomodal therapy has certain therapeutic effects, it is often accompanied by serious side effects or restrictive factors, such as low efficiency, easy degradation, lack of targeting, drug resistance, and so on.^[^ ^218^, ^219^, ^220^, ^221^, ^222^ ^]^ Consequently, bi‐modal and multimodal therapies are on the rise. The optical and electronic properties of BP make PTT the core of all combination therapies, as it can remedy constraints in the development of other therapies: 1) PTT‐induced hyperthermia can promote the cellular uptake of chemotherapy drugs, improve targeting, and prolong drug release time; 2) PTT can generate increased endocytosis of photosensitizers; more importantly, it accelerates the flow of blood around cancer cells and increases oxygen levels, thereby addressing one of the biggest drawbacks of PDT; 3) PTT increases the uptake of genes by tumor cells and accelerates gene release from DDSs via hyperthermia. Other therapies can offset problems with PTT, and this mutual promotion mechanism has led to the adoption of more and more combination therapies.

But despite these successes, several key challenges still need to be tackled to further advance PTT's biological and biomedical applications. First, when multiple modes of combination therapy are used, order and time intervals become the most important factors to consider, not only increasing the burden of administration for patients but also reducing optimal treatment. The advantage of multiple modes is often simultaneous rather than sequential function. But sometimes, the application of multiple treatments can exceed a patient's maximum tolerance. So optimizing the efficiency of combination therapy depends on using low doses and short irradiation times to achieve maximum anticancer effect and avoid the side effects caused by high doses and extended irradiation. In this context, the simultaneous use of multiple therapies at a single wavelength is a feasible strategy, such as the simultaneous stimulation of PTT and drug release‐induced chemotherapy, or the conversion of partial NIR to visible light by material design to stimulate both phototherapies at the same time. We also need to think about expanding the range of combination therapies. Because tumors tend to be located inside the body, it is necessary to coordinate DDSs with penetrating therapies, such as radiation. In addition, when treating certain malignant or metastatic tumors, the body's immune system can be stimulated through immunotherapy, and a self‐cleaning treatment can be carried out by strengthening the immune system.

Good biocompatibility is one of the most indispensable features of biomaterials, and it is the most prominent and attractive property of BP. Various studies have been conducted to verify the biocompatibility of BP through in vitro/in vivo experiments using pure BP platforms, modified BP, and DDSs loaded with drugs. The most commonly used test is MTT, measuring hemolysis and pathological changes in the blood and organs in vivo after injection. But in most of the reported studies, evaluation tests have only included simple laboratory testing (in vitro or in vivo). In reality, only a tiny fraction of these DDSs have a realistic possibility of advancing to the clinical trial stage, and actual clinical applications are still a long way from being tested. More importantly, solving the biosafety concerns generated by introducing nanomaterials into the body would have very high clinical value. Understanding the interactions between DDSs and substances in the body is key for achieving clinical transformation and biosafety; for example, the uncontrolled absorption of proteins often leads to the formation of a shell out of the DDSs after they enter the body, which reduces the efficiency of treatment.^[^ ^258^, ^259^ ^]^ We also need a greater understanding of the specific killing of tumor cells rather than normal cells, and the mechanisms of tumor death, to further the application and optimization of DDSs in biomedicine. Kong et al., for example, specifically explored a selective cancer cell death mechanism and proposed the following process: Selectively increased levels of phosphate anions in cancer cells → lipid peroxidation↑→ superoxide dismutase (SOD)↓→ ROS↑→ selective cancer cell death. They also identified a certain killing dosage range, which provides fundamental guidance for the safe biomedical applications of BP‐based materials.^[^ ^258^ ^]^ Besides, although BP materials are usually regarded as biodegradable and biocompatible, we need systematic investigation of their long‐term effects, and the in vivo observation period should be extended to at least half a year to yield pertinent assessment reports. To meet the strict requirements of preclinical experiments and reflect actual therapeutic effects, future evaluations should be extended to large‐animal models (e.g., dogs and monkeys) before translation of the technologies into clinical settings.

## Conflict of Interest

The authors declare no conflict of interest.
